# Excited state dynamics for visible-light sensitization of a photochromic benzil-subsituted phenoxyl-imidazolyl radical

**DOI:** 10.3762/bjoc.15.229

**Published:** 2019-10-04

**Authors:** Yoichi Kobayashi, Yukie Mamiya, Katsuya Mutoh, Hikaru Sotome, Masafumi Koga, Hiroshi Miyasaka, Jiro Abe

**Affiliations:** 1 Department of Applied Chemistry, College of Life Sciences, Ritsumeikan University, 1-1-1 Nojihigashi, Kusatsu, Shiga 525-8577, Japan; 2 Department of Chemistry, School of Science and Engineering, Aoyama Gakuin University, 5-10-1 Fuchinobe, Chuo-ku, Sagamihara, Kanagawa 252-5258, Japan; 3 Division of Frontier Materials Science and Center for Promotion of Advanced Interdisciplinary Research, Graduate School of Engineering Science, Osaka University, Toyonaka, Osaka 560-8531, Japan

**Keywords:** biradical, energy transfer, photochromism, sensitizer, transient absorption spectroscopy

## Abstract

Visible-light sensitized photoswitches have been paid particular attention in the fields of life sciences and materials science because long-wavelength light reduces photodegradation, transmits deep inside of matters, and achieves the selective excitation in condensed systems. Among various photoswitch molecules, the phenoxyl-imidazolyl radical complex (PIC) is a recently developed thermally reversible photochromic molecule whose thermal back reaction can be tuned from tens of nanoseconds to tens of seconds by rational design of the molecular structure. While the wide range of tunability of the switching speed of PIC opened up various potential applications, no photosensitivity to visible light limits its applications. In this study, we synthesized a visible-light sensitized PIC derivative conjugated with a benzil unit. Femtosecond transient absorption spectroscopy revealed that the benzil unit acts as a singlet photosensitizer for PIC by the Dexter-type energy transfer. Visible-light sensitized photochromic reactions of PIC are important for expanding the versatility of potential applications to life sciences and materials science.

## Introduction

Photochromism, which is defined as the reversible transformation of a chemical species between two structural isomers by light, has been extensively studied over decades [1–4]. Recently, visible-light sensitized photochromic materials have been paid particular attention in the fields of life sciences and materials science because long-wavelength light reduces photodegradation, transmits deep inside of matters, and achieves the selective excitation in condensed systems [5–12]. General strategies for the sensitization of the photochromic reactions to visible light are to extend the π-conjugation and to utilize photosensitizers. Especially, triplet photosensitizers, which form the triplet state of a molecule by the triplet–triplet energy transfer, have been frequently used in photoresists, photodynamic therapy, and photocatalysts because the lowest triplet excited (T_1_) state can be formed by light whose energy is smaller than that of the optically active transition [13–16]. However, photochromic reactions of some systems do not proceed via the T_1_ state. For example, it was reported that the photochromic reaction of hexaarylbiimidazole (HABI), which is a well-known radical-dissociation-type photochromic molecule [17–20], is not sensitized by triplet photosensitizers [21–23]. On the other hand, it was reported that singlet photosensitizers effectively sensitize the photochromic reaction of HABI to the visible light [21,23]. While the S_0_–S_1_ transition of HABI is located at the visible-light region, the transition is optically forbidden. Therefore, the photochromic reaction of HABI without singlet photosensitizers occurs via the S_0_–S_n_ transition, which is located at the UV region. On the other hand, singlet photosensitizers efficiently transfer the visible-light energy to the optically inactive S_1_ state of HABI, and thus the photochromic reaction of HABI proceeds with visible light.

**Scheme 1:**
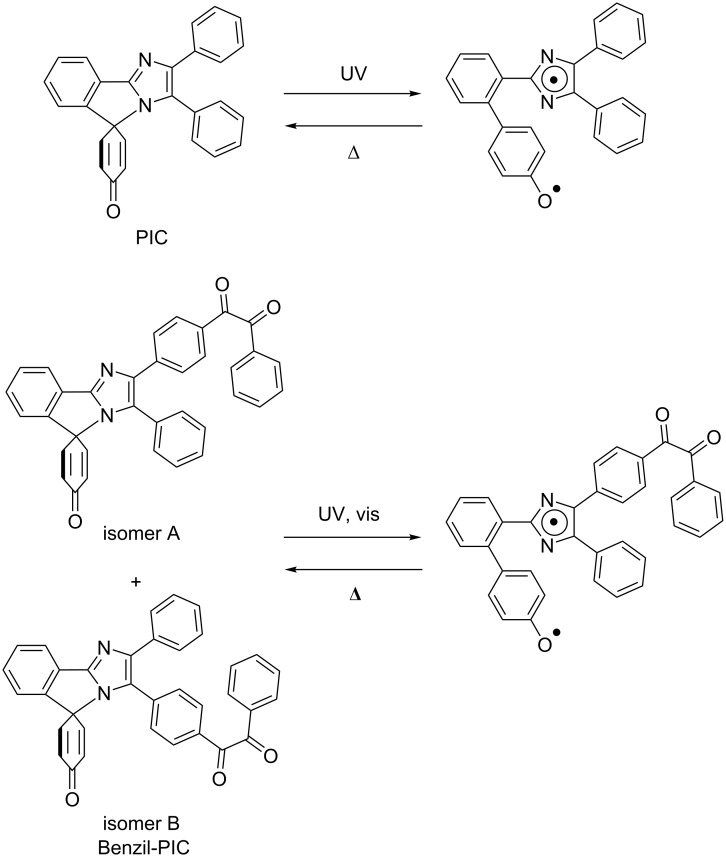
Photochromic reaction schemes of (a) PIC and (b) Benzil-PIC.

The phenoxyl-imidazolyl radical complex (PIC, Scheme 1) is one of the recently developed rate-tunable T-type photochromic compounds which reversibly generate an imidazolyl radical and a phenoxyl radical (biradical form) in a molecule upon UV light irradiation [24]. The great advantage of PIC is the tunability of the thermal back reaction from tens of nanoseconds to tens of seconds by simple and rational molecular design [25]. The wide ranges of thermal back reactions of photoswitches expand the potential applications of photochromic materials such as to dynamic holographic display [26–28], switchable fluorescent markers [29–31], and anticounterfeit inks. However, PIC is photosensitive only in the UV region, which limits the application fields. It was reported that the S_0_–S_1_ transition of PIC is optically forbidden and is located at the visible-light region as similar to that of HABI [32]. It is expected that the photochromic reaction of PIC occurs via the optically forbidden S_1_ state as similar to other radical dissociation-type photochromic molecules such as HABI and pentaarylbiimidazole (PABI) [33–35]. Therefore, if we could substitute a singlet photosensitizer unit to PIC, the visible-light sensitivity could be achieved by singlet–singlet energy transfer. The visible-light sensitization of PIC expands the versatility of the rate-tunable photoswitches of PIC systems.

In this study, we synthesized a novel PIC derivative conjugated with a visible-light photosensitizer (Benzil-PIC, Scheme 1) and investigated the excited state dynamics. We used a benzil framework as a photosensitizer unit because aryl ketones have been widely used as visible-light photosensitizers [36]. While most of aryl ketones were used as triplet photosensitizers, the benzil unit in the present study acts as a singlet photosensitizer. The detail of the sensitization processes was investigated by wide ranges of time-resolved spectroscopies.

## Results and Discussion

### Steady-state absorption spectra

**Figure 1:**
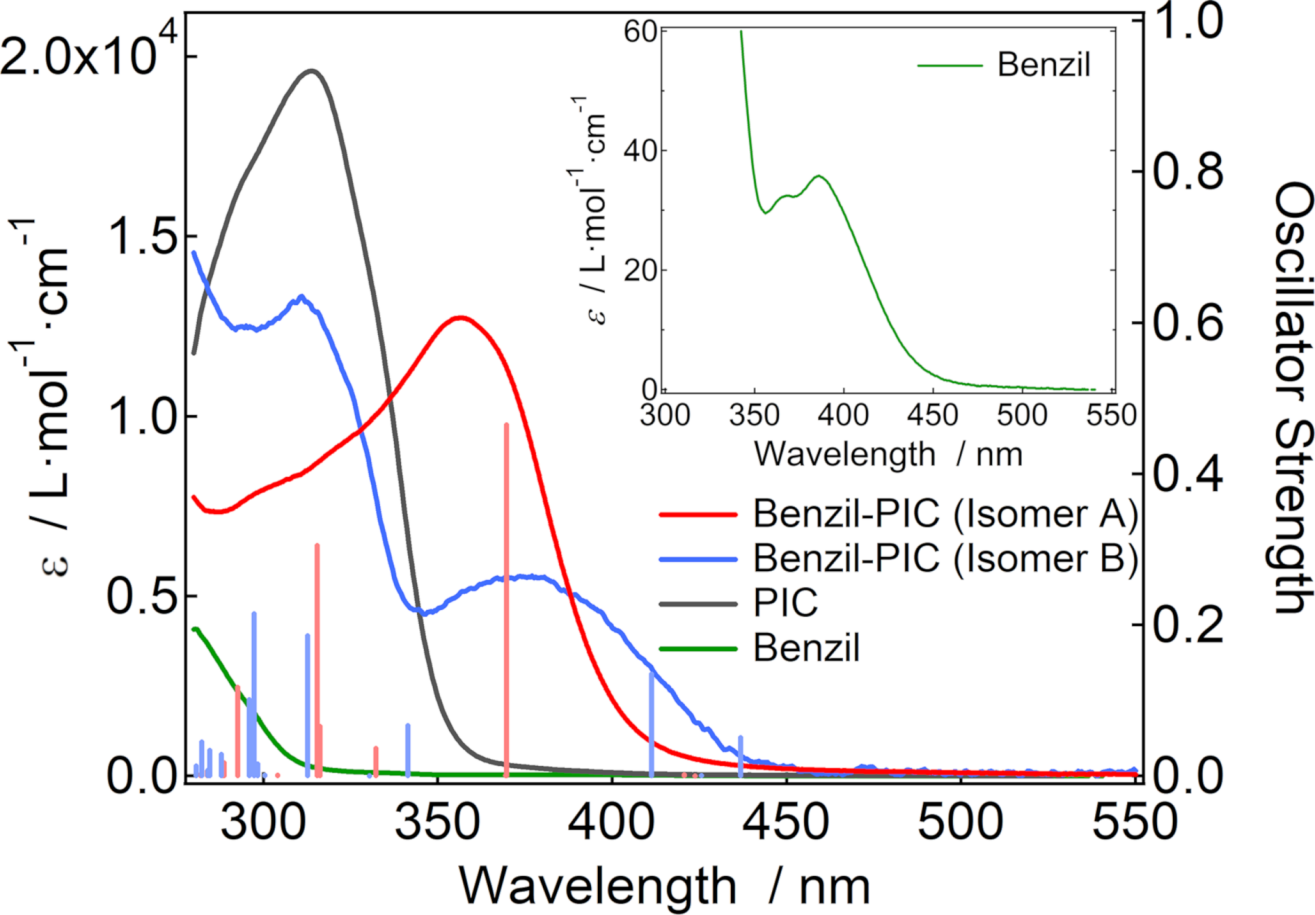
Absorption spectra of PIC, benzil, and the two isomers of Benzil-PIC in benzene at 298 K. The inset shows the magnified absorption spectrum of benzil in benzene. The calculated spectra (MPW1PW91/6-31+G(d,p)//M05-2X/6-31+G(d,p) level of the theory) of two isomers of Benzil-PIC are shown as the vertical lines.

The synthetic procedure of Benzil-PIC is described in the Experimental part. Benzil-PIC has two structural isomers (isomer A and isomer B) as shown in Scheme 1. These isomers were separated by high-performance liquid chromatography (HPLC), and each isomer was characterized by steady-state absorption spectra and time-dependent density functional theory (TDDFT) calculations as shown below. Figure 1 shows the steady-state absorption spectra of the two isomers of Benzil-PIC and PIC in benzene at 298 K.

While the absorption of PIC appears only at wavelength shorter than 350 nm, those of the two isomers of Benzil-PIC are extended to the visible-light region. The simulated absorption spectra by TDDFT calculations (MPW1PW91/6-31+G(d,p)//M05-2X/6-31+G(d,p) level of the theory) are also shown as the vertical lines in Figure 1. The simulated absorption spectra well explain the experimental absorption spectra of the two isomers. Therefore, the absorption spectra of isomers A and B were assigned as shown in Figure 1. The absorption band at 357 nm of isomer A is assigned to the electronic transition from the molecular orbital distributed around the triphenylimidazole unit (highest occupied molecular orbital: HOMO) to that around the benzil unit (the second lowest unoccupied molecular orbital: LUMO+1) (Figure S14, Supporting Information File 1). On the other hand, the absorption band at 375 nm of isomer B is assigned to the electronic transition from the molecular orbital distributed around the triphenylimidazole unit (HOMO) to that around the benzil unit and the phenoxyl unit (mainly the lowest unoccupied molecular orbital: LUMO and LUMO+1, Figure S15, Supporting Information File 1). While the HOMOs of isomer A and isomer B are very similar, the LUMO and LUMO+1 of isomer B are more delocalized than the LUMO+1 of isomer A, suggesting that the LUMO and LUMO+1 levels of isomer B are lower than those of isomer A. This would be the most plausible reason why isomer B has an absorption band at the longer wavelength than isomer A.

PIC generates the biradical species upon UV-light irradiation and shows the broad transient absorption spectrum over the visible- to near infrared-light regions. The half-life of the thermal back reaction of the biradical in benzene is 250 ns (the lifetime is 360 ns) at 298 K. To investigate the difference in the photochromic properties between two isomers of Benzil-PIC, we measured the absorption spectra and nanosecond-to-microsecond transient absorption dynamics of isomer A in benzene upon repeated irradiation of 355 nm nanosecond laser pulses (355 nm, 7 mJ pulse^−1^, Figure S8a, Supporting Information File 1). The absorption band at 357 nm of isomer A gradually decreases upon irradiation of the nanosecond laser pulses and the absorption edge alternatively shifts to the longer wavelength. It indicates that the irradiation of the UV pulse induces the photochromic reactions (breaking of the C–N bond) and interconverts between isomer A and isomer B. The system reaches the photostationary state (PPS) within 696 shots of the laser pulses. The ratio of isomer A and isomer B is estimated to be 22:78 by the curve fitting of the absorption spectrum at the PPS with those of pure isomer A and isomer B (Figure S9, Supporting Information File 1). Figure S8b (Supporting Information File 1) shows the nanosecond-to-microsecond transient absorption dynamics of isomer A probed at 650 nm under repeated irradiation with the 355 nm nanosecond laser pulses at 298 K. While the transient absorption dynamics of isomer A accumulated by 8 shots are slightly fluctuated most probably because of the low signal-to-noise ratio, the decay kinetics do not change by repeated irradiation with UV-light pulses. It shows that both isomers generate the same biradical form by UV-light irradiation as shown in Scheme 1, indicating that the excited state dynamics of the two isomers of Benzil-PIC after the bond breaking are identical. Therefore, the mixture solution of the two isomers was used for further time-resolved spectroscopic measurements.

### Nanosecond-to-microsecond transient absorption spectra

**Figure 2:**
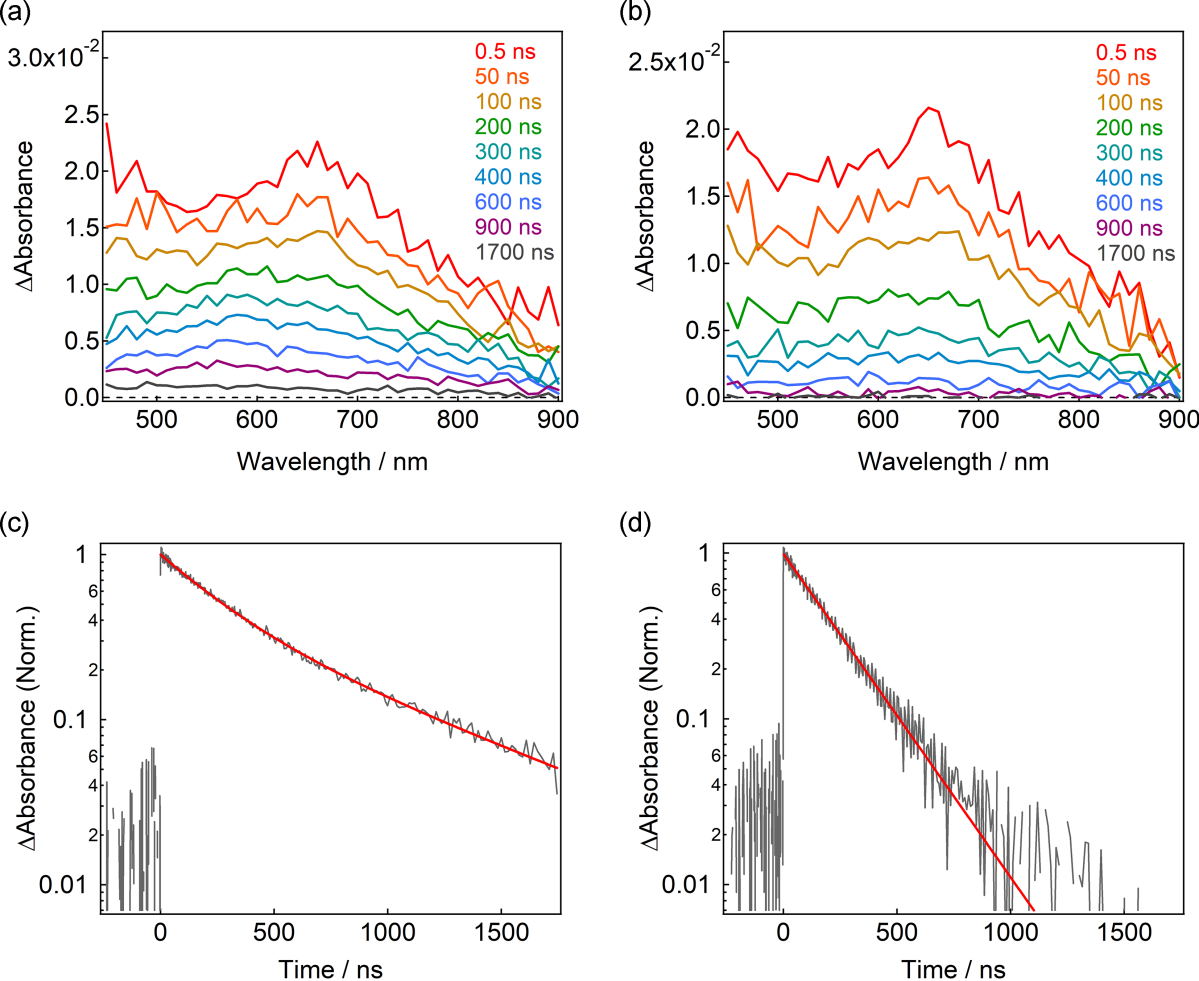
Nanosecond-to-microsecond transient absorption spectra of Benzil-PIC in benzene under (a) argon and (b) air at room temperature excited with a 355 nm picosecond laser pulse (30 μJ pulse^−1^). Decay profiles of the transient species of Benzil-PIC in benzene probed at 590 nm under (c) argon and (d) air at the same conditions.

To investigate the photochromic properties of Benzil-PIC, the nanosecond-to-microsecond transient absorption measurements were conducted by the randomly interleaved pulse train (RIPT) method [37]. Figure 2a shows the transient absorption spectra of Benzil-PIC in benzene (2.9 × 10^−4^ M) under argon atmosphere at room temperature excited with a 355 nm picosecond laser pulse (pulse duration = 25 ps, intensity = 30 μJ pulse^−1^).

At 0.5 ns after the excitation, two broad transient absorption bands are observed at 660 and <450 nm. The spectral shape is more or less similar to that of the biradical form of PIC [24], indicating Benzil-PIC generates the biradical by 355 nm light irradiation. The transient absorption spectra gradually decay with a time scale of hundreds of nanoseconds and another absorption band at 580 nm remains after 900 ns. The transient absorption dynamics at 590 nm was fitted with a biexponential decay function and the lifetimes are estimated to be 260 and 820 ns (Figure 2c). On the other hand, while the transient absorption spectra of Benzil-PIC in benzene under air show the same transient absorption spectrum as under argon at 0.5 ns, the transient absorption band at 580 nm is not observed in the time scale of microseconds. The transient absorption dynamics at 590 nm can be fitted with a single exponential decay function and the lifetime is 220 ns (Figure 2d), which is almost identical to that of the fast decay component under argon atmosphere. Because the transient absorption spectrum at 0.5 ns is similar to that of PIC and because the fast decay component does not depend on the molecular oxygen, the fast and slow decay components can be assigned to the biradical form generated by the C–N bond breaking and the T_1_ state of Benzil-PIC, respectively. It is worth mentioning that the T_1_ state of Benzil-PIC would be formed by some portions of the S_1_ of the benzil unit where the energy transfer did not occur to the PIC unit (discussed below).

### Femtosecond-to-nanosecond transient absorption spectra

**Figure 3:**
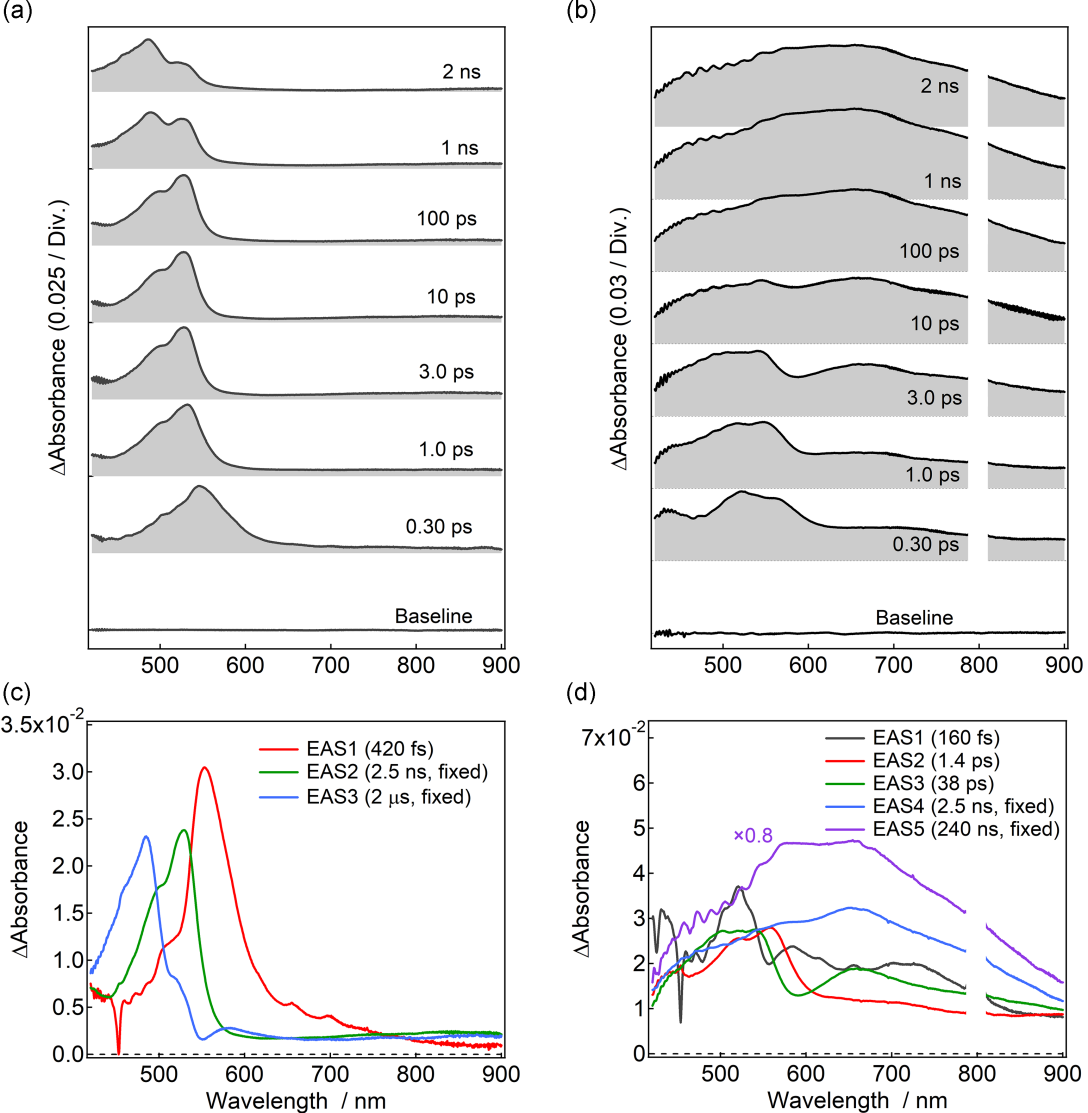
Femtosecond-to-nanosecond transient absorption spectra of (a) benzil and (b) Benzil-PIC (right) in benzene at room temperature excited at 400 nm (100 nJ pulse^−1^, ≈100 fs laser pulse). The evolution associated spectra (EAS) of (c) benzil and (d) Benzil-PIC in benzene at the same experimental conditions. The signal around 800 nm of Benzil-PIC was omitted because of the second-order diffraction of the excitation pulse.

To investigate the sensitization process by the benzil unit of Benzil-PIC in detail, we performed femtosecond transient absorption measurements using a 400 nm excitation pulse. The instrumental response function is ≈170 fs. It is noted that the excitation wavelength for femtosecond transient absorption spectroscopy (400 nm) is slightly different from that for nanosecond transient absorption spectroscopy (355 nm). The difference may affect the ratio of isomer A and isomer B at the photostationary state (PSS) and initial relaxation kinetics at sub-picosecond time scales. Benzil was used for a reference sample. Figure 3a shows the time evolution of the transient absorption spectra of benzil in benzene (6.8 × 10^−2^ M).

At 0.3 ps after the excitation, a transient absorption band is observed at 546 nm. The transient absorption band continuously shifts to 531 nm and a shoulder is observed at 500 nm. It was reported that the spectral shift of the transient absorption spectra of benzil at the sub-picosecond time scale was assigned to the structural change from the skewed structure to the planar structure [38]. Solvent and vibrational relaxations would also take place in this time scale. After the rapid spectral shift, the transient absorption spectra are preserved until 100 ps. This signal can be assigned to the excited state absorption from the lowest vibrational level of the S_1_ state. The transient absorption band at 531 nm gradually decreases with a time scale of nanoseconds and another transient absorption band appears at 485 nm. The transient absorption band at 485 nm was assigned to the T_1_ state according to previous studies [39–41]. The quantum yield of the formation of the triplet excited state was reported as 92% [42], indicating that most of the S_1_ state is converted to the T_1_ state in benzil.

Figure 3b shows the transient absorption spectra of Benzil-PIC in benzene (2.2 × 10^−3^ M) excited at 400 nm with a femtosecond laser pulse. The signal around 800 nm was omitted because it was perturbed by the second order diffraction of the excitation pulse around 400 nm. At 0.3 ps after the excitation, two transient absorption bands are observed at 520 and 563 nm, which are most probably assigned to the transient absorption of the benzil unit of Benzil-PIC. The absorption is slightly shifted to the red as compared to those of benzil probably due to the extended π-conjugation of the benzil unit connected to the PIC unit. The two peaks continuously shift to the shorter wavelength (503 and 543 nm, respectively) with a time scale of picoseconds as similar to that of benzil, which supports that these bands are originated from the benzil unit. In addition to the two bands, a broad absorption band over the visible-light region is also observed at 0.3 ps. Because the spectral band shape of this absorption band is similar to that observed in Figure 2, this absorption band is ascribable to the biradical form of PIC, which was directly excited at 400 nm and underwent the rapid radical formation in the sub-picosecond time range. The instantaneous formation of the biradical form under these excitation conditions suggests that a peak at ≈430 nm at 0.3 ps would be most probably assigned to the S_1_ state of the PIC unit. In addition to this rapid appearance of the biradical form, the gradual increase of the absorption due to the biradical is observed in picoseconds to tens of picoseconds region, together with the decay of the S_1_ state of the benzil unit. This slow process of the biradical formation indicates the energy transfer from the benzil unit to the PIC unit. The amplitude of the increased biradical form with a time scale of tens of picoseconds is larger than the instantaneously generated biradical form at the early time scale, indicating that the energy transfer process is dominant for the photochromic reaction of Benzil-PIC under the excitation with 400 nm. In the nanoseconds time region, the absorption around 580 nm slightly increases with a time scale of nanoseconds.

To elucidate the details of the reaction dynamics, we performed global analyses with singular value decomposition (SVD) with the Glotaran program (http://glotaran.org) [43]. We tentatively used the three-state sequential kinetic model for benzil (Equation 1) and the five-state sequential kinetic model for Benzil-PIC (Equation 2) convolved with Gaussian pulse. The detail of the SVD analyses are shown in Supporting Information File 1.

[1] [A]→[B]→[C]→ground state

[2] [A]→[B]→[C]→[D]→[E]→ground state

The evolution associated spectra (EAS) thus obtained indicate the resolved transient absorption spectra into each component of the kinetic models. Because the time window of our measurements was limited to 2 ns, it was difficult to determine the time constant of nanosecond time scale exactly. Therefore, the lifetimes of the intersystem crossing (ISC) of benzil and the benzil unit of Benzil-PIC were fixed to a reported value of benzil (2.5 ns) [44]. The lifetime of the T_1_ state of benzil was fixed to 2.0 μs according to the nanosecond-to-microsecond transient absorption spectroscopy. In the benzil system, time constants of three EAS are revealed to be 420 fs, 2.5 ns (fixed), and 2.0 μs (fixed), respectively (Figure 3c). Each EAS species (A to E in the Equation 1 and Equation 2) is denoted as EAS1 to EAS5 in the order of the time constants as shown in Figure 3c and Figure 3d. The fastest time constant of benzil reflects the structural change from the skewed structure to the planar structure and solvent and vibrational relaxations. However, it should be noted that the lifetime of 420 fs is the apparent lifetime because the conformational change from the skewed to the planar structure at sub-picosecond time scale induces the continuous spectral shift. Because the present SVD global analyses do not consider the continuous spectral shift, it is difficult to extract the exact time constant at the early stage of the transient absorption spectra. The EAS with time constants of 2.5 ns and 2.0 μs are safely assigned to the absorption spectra of the S_1_ and the T_1_ states, respectively, because of the similarity of the spectra to those reported previously [39–40].

In the Benzil-PIC system, the time constants of five EAS were obtained to be 160 fs, 1.4 ps, 38 ps, 2.5 ns (fixed), and 240 ns (fixed), respectively (Figure 3d). EAS1 has 4 peaks located at 430, 520, 582, ≈710 nm, respectively. The absorption bands at 430 and ≈710 nm are ascribable to the S_1_ state of the PIC unit and the biradical generated instantaneously, respectively. It indicates that the biradical was also formed by the direct excitation of the PIC unit with 400 nm light. The spectral evolution from EAS1 (160 fs, grey line in Figure 3d) to EAS2 (1.4 ps, red line in Figure 3d) shows the C–N bond cleavage of the PIC unit and the spectral shift due to the benzil unit (from 582 nm to 556 nm). In PABI, which is a similar photochromic molecule to PIC, it was reported that the C–N bond fission occurs with the time constant of 140 fs and the broad absorption assigned to the biradical form was formed with a time constant of ≈2 ps [45]. The similarity of the time constant of the bond breaking to that of EAS1 supports that the C–N bond is cleaved by the direct excitation of the PIC unit. The spectral evolution from EAS2 (1.4 ps) to EAS3 (38 ps, green in Figure 3d) shows the continuous spectral shift due to the benzil unit and the increase in the absorption due to the biradical form (660 nm). Because the continuous spectral shift due to the benzil unit is still observed in EAS2 (1.4 ps), it is suggested that the structural change of the benzil unit of Benzil-PIC is somehow slightly decelerated as compared to that of benzil (420 fs). However, it should be mentioned that it was difficult to resolve the structural change of the benzil unit and the formation process of the PIC unit by the present SVD analysis.

The spectral evolution from EAS3 (38 ps) to EAS4 (2.5 ns, fixed, blue line in Figure 3d) shows the decay of the S_1_ state of the benzil unit and the alternative increase in the biradical form of the PIC unit. This result clearly shows that the energy of the S_1_ state of the benzil unit is used for the photochromic reaction of the PIC unit. It is important to note that the S_0_–S_1_ transition energy of PIC, which is optically forbidden, was reported to be 2.8 eV (≈440 nm) [32]. These results suggest that the energy transfer occurs from the S_1_ state of the benzil unit to the ground state of the PIC unit with the time constant of 38 ps. Since the bond-breaking process from the S_1_ state of the PIC unit would be much faster than this time scale (hundreds of femtoseconds), the time constant of 38 ps reflects the singlet–singlet energy transfer process from the benzil unit to the PIC unit. It should be noted that the fluorescence quantum yield of benzil was quite low (<0.001) [43] and the PIC unit has no absorption in the emission wavelength of the benzil. Accordingly, the effective energy transfer by the Förster mechanism is not plausible. The energy transfer of the 38 ps time constant is probably due to the Dexter mechanism at weak or very weak coupling regimes owing to the overlap of the wave functions of the benzil and the PIC units in the excited state.

The spectral evolution from EAS4 (2.5 ns, fixed) to EAS5 (240 ns, fixed, purple line in Figure 3d) shows the increase in the absorption around 580 nm. Although both lifetimes of EAS4 and EAS5 are longer than the measured time window (≈2 ns), the spectral difference around 580 nm at 10–100 ps and that at nanosecond time scales enable to resolve these spectra. The increased absorption band is similar to the transient absorption band assigned to the T_1_ state of Benzil-PIC (Figure 2a). It indicates that the spectral evolution over nanosecond time scale is ascribable to ISC of the benzil unit. It should be noted, however, that this slow rise of the T_1_ state of the benzil unit by ISC indicates that some portions of the benzil unit do not undergo the effective energy transfer to the PIC unit because the S_1_ state of the benzil was deactivated with the time constant of 38 ps. Although the clear mechanism is not yet elucidated at the present stage of the investigation, the reason for the two relaxation pathways (energy transfer and ISC) from the S_1_ state of the benzil unit of Benzil-PIC might be due to the difference in the mutual orientation of benzil and PIC units including the structural isomers (isomer A and isomer B). As was discussed above, the energy transfer is due to the overlap of the wave function of the both units, of which mechanism might be sensitive to the difference in the mutual orientation.

### Effect of triplet–triplet energy transfer

**Figure 4:**
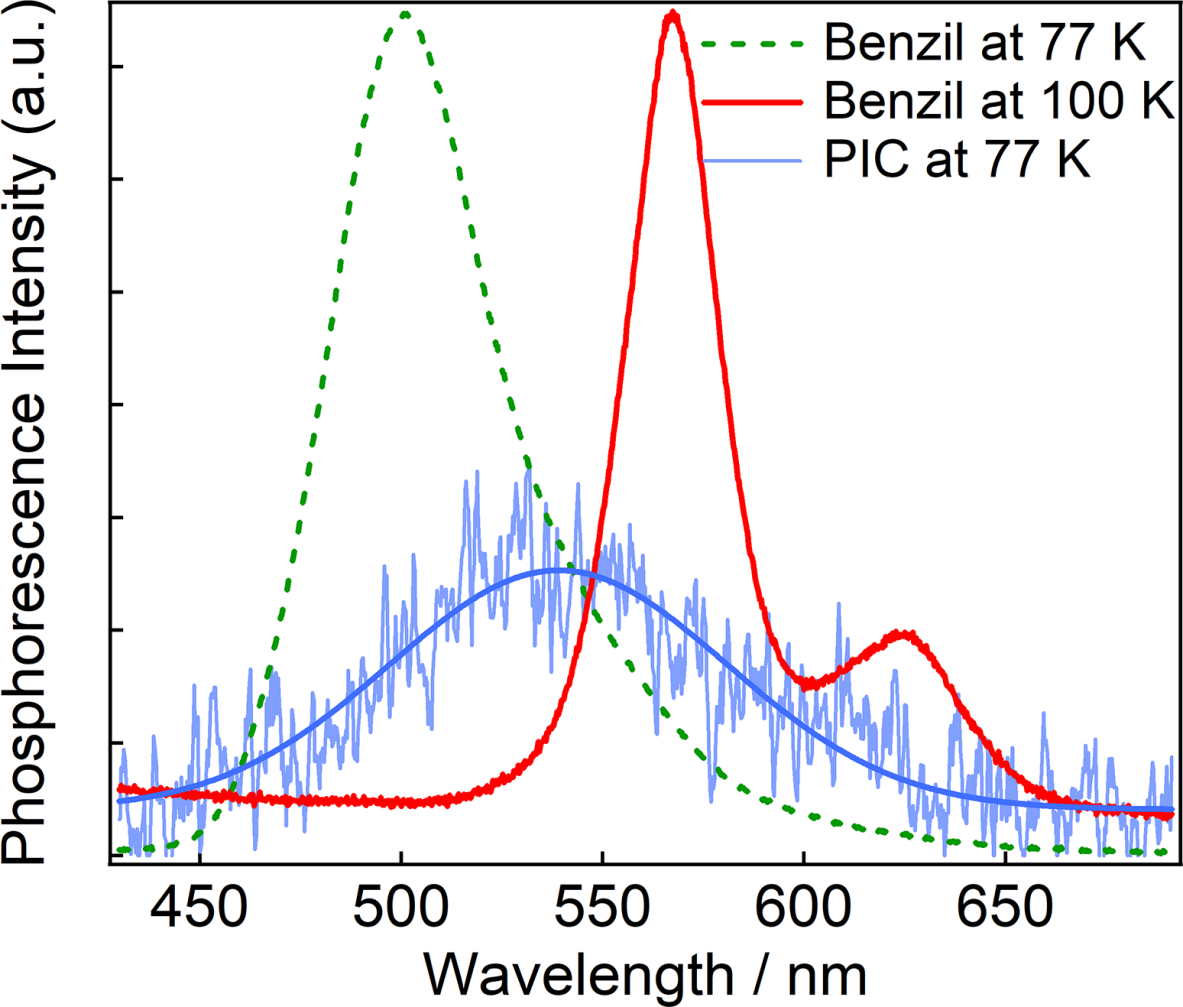
Phosphorescence spectra of benzil at 77 K and 100 K and that of PIC at 77 K in EPA. A blue solid line on the phosphorescence spectra of PIC is the Gaussian fitting line.

Ultrafast spectroscopy revealed that the benzil unit acts as a singlet photosensitizer for Benzil-PIC by the Dexter-type energy transfer. It was reported that benzil was often used as a triplet photosensitizer because the quantum yield for the T_1_ state formation is 92% [42]. To investigate the possibility for the triplet–triplet energy transfer process in Benzil-PIC, we performed two experiments. Firstly, we measured the phosphorescence spectra of benzil and PIC in EPA (diethyl ether/isopentane/ethanol 5:5:2) at low temperature to estimate the energy levels of the T_1_ states of benzil and PIC. In the conventional emission measurement setups at low temperature, both fluorescence and phosphorescence are observed upon irradiation of excitation light. To extract the phosphorescence spectra, the excitation light (continuous wave laser, 355 nm, 1 mW) was chopped at 1 Hz and the afterglow emission under blocking the beam was accumulated as the phosphorescence spectra. Figure 4 shows the phosphorescence spectra of benzil in EPA at 77 and 100 K.

While the phosphorescence spectrum of benzil at 77 K is broad and observed at 500 nm, that at 100 K becomes sharper and the peak is shifted to 567 nm with a vibrational fine structure at 625 nm. The spectral shift with the increase in temperature is most probably due to the rigidity of the environment of molecules. At 77 K, it is expected that the solvent is too rigid for benzil to change the conformation in the excited state, namely, the conformation of benzil is fixed to the skewed conformation. On the other hand, it is expected that the increase in the temperature to 100 K softens the rigid matrix and allows the benzil to form the planar conformation at the T_1_ state. The energy level of the T_1_ state of benzil was estimated from the phosphorescence at 100 K because the T_1_ state of benzil in solution forms the planar conformation. The energy level of the T_1_ state was determined by an edge of the high energy side of the phosphorescence, where a tangent line crosses the *x*-axis. The energy level of the T_1_ state of benzil is estimated to be 53 kcal mol^−1^, which is consistent with a reported value (53.7 kcal mol^−1^) [38]. On the other hand, the phosphorescence of PIC was only observed at 77 K and the signal is very weak. Because the conformation of PIC is relatively rigid, we tentatively estimated the T_1_ state energy level from the phosphorescence at 77 K. The T_1_ state energy level of PIC is estimated to be 63 kcal mol^−1^. It suggests that the T_1_ state energy level of benzil is slightly lower than that of PIC.

Moreover, the triplet photosensitization was examined by the microsecond transient absorption measurements of the mixture solution of benzil and PIC in benzene (3.7 × 10^−3^ M and 2.8 × 10^−5^ M for benzil and PIC, respectively). A 450 nm excitation pulse was used to selectively excite benzil. The transient absorption dynamics of the mixture solution of benzil and PIC probed at 500 nm is identical to that of benzil, which is assigned to the T_1_ state (Figure S13, Supporting Information File 1). It indicates that the triplet–triplet energy transfer is negligible between the benzil and PIC units. The plausible reason for the negligible triplet–triplet energy transfer is the lower energy level of the T_1_ state of the benzil unit than that of the PIC unit.

## Conclusion

**Figure 5:**
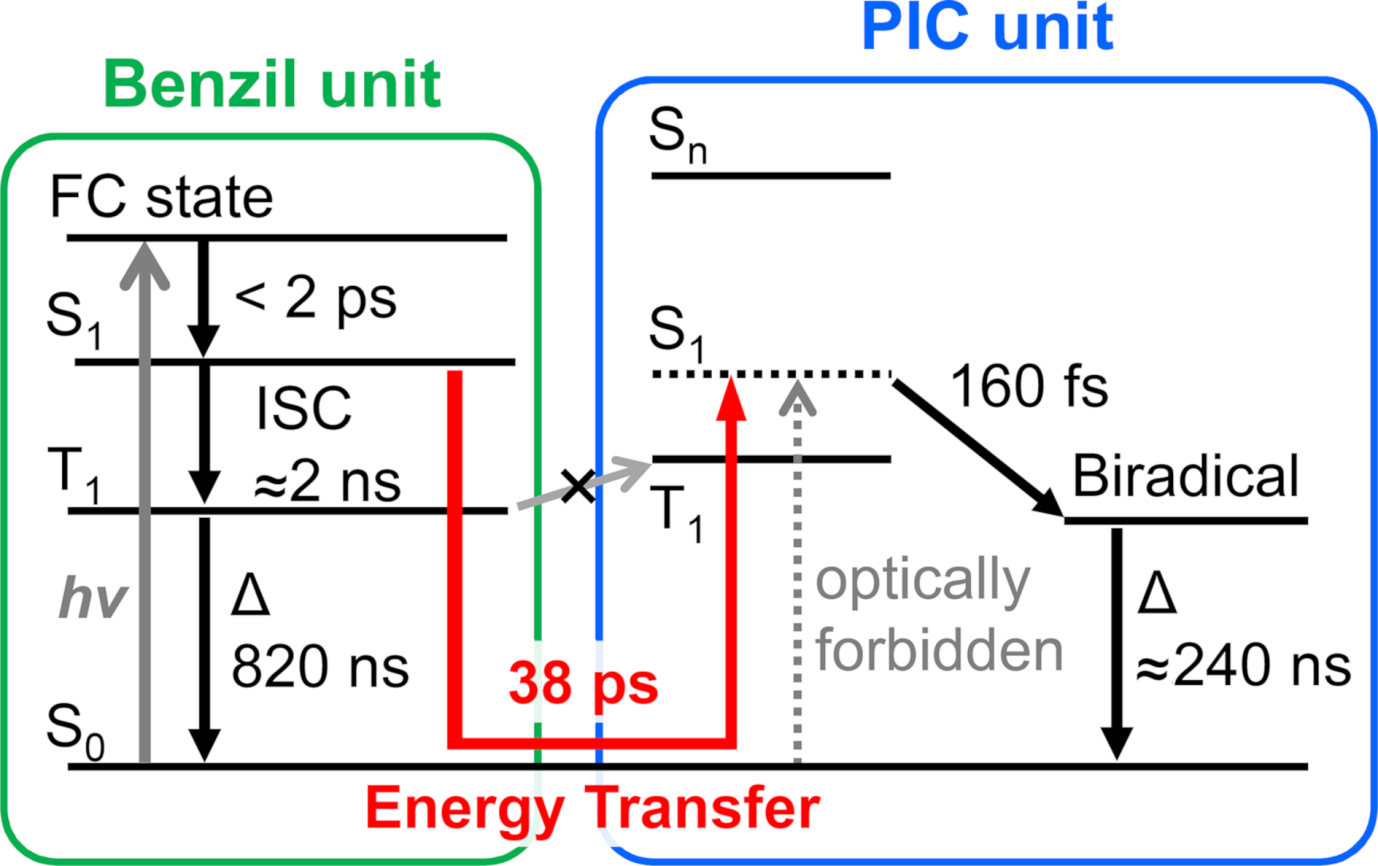
Energy diagram of the visible-light sensitized photochromic reaction of Benzil-PIC.

Figure 5 describes the energy diagram for the photochromic reaction of Benzil-PIC.

While PIC absorbs light of wavelength only shorter than 350 nm, the introduction of the benzil unit extends the photosensitivity of the photochromic reaction to the visible-light region. When Benzil-PIC absorbs visible light, the conformation of the benzil unit, which is the skewed structure in the ground state, quickly changes to the planar structure with a time scale of picoseconds and the S_1_ state of the benzil is formed. While the photochromic reaction partly proceeds via the direct excitation of the PIC unit, most of the photochromic reaction is induced via the Dexter-type singlet–singlet energy transfer from the benzil to the PIC units with the time constant of 38 ps. The triplet photosensitization does not occur in Benzil-PIC most probably because the triplet energy level of the PIC unit is higher than that of the benzil unit. The clarification of the visible-light sensitization mechanism of PIC is important for expanding the versatility of potential applications of PIC in life and materials sciences.

## Experimental

### Synthetic procedures

All reactions were monitored by thin-layer chromatography carried out on 0.2 mm E. Merck silica gel plates (60F-254). Column chromatography was performed on silica gel (Silica Gel 60N (spherical, neutral), 40–50 μm, Kanto Chemical Co., Inc.). ^1^H NMR spectra were recorded at 400 MHz on a Bruker AVANCE III 400 NanoBay. DMSO-*d*_6_ and CDCl_3_ were used as deuterated solvents. Mass spectra (ESI-TOF-MS) were measured by using a Bruker micrOTOFII-AGA1. All reagents were purchased from TCI, Wako Co. Ltd., Aldrich Chemical Company, Inc. and Kanto Chemical Co., Inc., and were used without further purification.

**Scheme 2:**
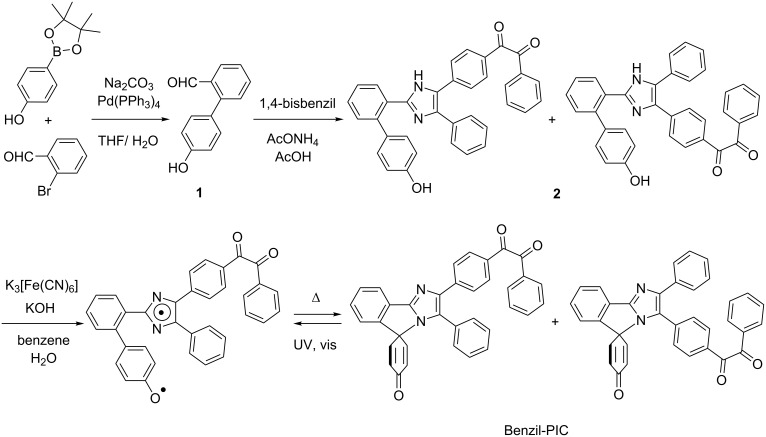
Synthetic procedure of Benzil-PIC (analogous to synthesis of PIC in [24]).

The synthetic procedure of Benzil-PIC is shown in Scheme 2. The synthetic procedure is analogous to that of PIC [24].

#### 4'-Hydroxy-[1,1'-biphenyl]-2-carbaldehyde (**1**)

Compound **1** was prepared according to a literature procedure [24].

#### 1-(4-(2-(4'-Hydroxy-[1,1'-biphenyl]-2-yl)-4-phenyl-1*H*-imidazol-5-yl)phenyl)-2-phenylethane-1,2-dione (**2**)

4’-Hydroxy-[1,1’-biphenyl]-2-carbaldehyde (**1**, 0.088 g, 0.44 mmol), 1,4-bisbenzil (0.176 g, 0.51 mmol) and ammonium acetate (0.240 g, 3.12 mmol) were stirred at 110 °C in acetic acid (2.7 mL) for 6 h. The reaction mixture was cooled and neutralized by aqueous NH_3_. The precipitate was filtered and washed with water. The crude product was purified by silica gel column chromatography (CH_2_Cl_2_/AcOEt 20:1 to 3:1), to give the desired product as a mixture of two structural isomers as a yellow solid, 0.0674 g (0.130 mmol, 29%). ^1^H NMR (DMSO-*d*_6_, 400 MHz) δ 12.21 (s, 1H, one structural isomer), 12.11 (s, 1H, one structural isomer), 9.42 (s, 1H, one structural isomer), 9.40 (s, 1H, one structural isomer), 7.92–7.90 (m, 4H, two structural isomers), 7.81–7.79 (m, 6H, two structural isomers), 7.72–7.69 (m, 5H, two structural isomers), 7.65–7.61 (m, 4H, two structural isomers), 7.53–7.50 (m, 3H, two structural isomers), 7.45–7.37 (m, 10H, two structural isomers), 7.33–7.30 (m, 4H, two structural isomers), 7.07–7.04 (m, 4H, two structural isomers), 6.73–6.69 (m, 4H, two structural isomers); ESI-TOF MS *m*/*z*: [M + H]^+^ calcd for C_35_H_24_N_2_O_3_: 521.1859691; found, 521.1836034.

#### Benzil-PIC

A solution of potassium ferricyanide (0.968 g, 2.94 mmol) and KOH (0.741 g, 13.2 mmol) in water (3.3 mL) was added to a suspension of **2** (70 mg, 0.14 mmol) in benzene (7.3 mL). After stirring for 3 h at room temperature, the resultant mixture was then extracted with benzene and the organic extract was washed with water and brine. After removal of solvents, the crude product was purified by silica gel column chromatography (AcOEt/hexane 2:3) to give the desired product as a yellow powder, 42 mg (0.081 mmol, 58%). Two structural isomers were separated by HPLC (eluent: CH_3_CN/H_2_O 7:3). ^1^H NMR (CDCl_3_, 400 MHz) (isomer A) δ 8.01 (d, *J* = 7.5 Hz, 1H), 7.97–7.94 (m, 2H), 7.84 (d, *J* = 8.6 Hz, 2H), 7.72 (d, *J* = 8.7 Hz, 2H), 7.66–7.48 (m, 5H), 7.39 (t, *J* = 7.0 Hz, 2H), 7.35–7.29 (m, 3H), 7.16 (d, *J* = 7.7 Hz, 1H), 6.57 (d, *J* = 10.0 Hz, 2H), 6.27 (d, *J* = 10.0 Hz, 2H), (isomer B) δ 7.98–8.02 (m, 3H), 7.89 (d, *J* = 8.4 Hz, 2H), 7.69 (t, *J* = 6.2 Hz, 1H), 7.57–7.48 (m, 8H), 7.40 (t, *J* = 7.6 Hz, 1H), 7.31–7.29 (m, 2H), 7.16 (d, *J* = 7.7 Hz, 1H), 6.64 (d, *J* = 10.0 Hz, 2H), 6.36 (d, *J* = 10.0 Hz, 2H); ESI-TOF MS *m*/*z*: [M + H]^+^ calcd for C_35_H_22_N_2_O_3_, 519.1703190; found, 519.1696883.

### Experimental setups

#### Steady-state measurements

Steady-state absorption spectra were measured with an UV-3600 Plus (SHIMADZU) at room temperature with 1 cm quartz cuvette. Phosphorescence spectra were measured by home-build millisecond time-resolved emission spectrometer at 77 K with nitrogen cryostat (OptistatDN2, Oxford instruments). Briefly, the cooled samples in EPA (diethyl ether/isopentane/ethanol 5:5:2) under argon atmosphere were excited with a 355-nm continuous wave (CW) laser (Genesis CX355 100SLM AO, Coherent) and the emission was detected by EMCCD (Newton DU920P-OE, Andor Technology). The excitation light was blocked with 1 Hz by an optical shutter (76992 and 6995, ORIEL) and the time evolution of the emission spectra was measured to separate the fluorescence and phosphorescence. The shutter was controlled by LabVIEW.

#### Nanosecond transient absorption measurements

The laser flash photolysis experiments were carried out with a TSP-2000 time resolved spectrophotometer system (Unisoku Co., Ltd.). A 10 Hz Q-switched Nd:YAG laser (Continuum Minilite II) with the third harmonic at 355 nm (pulse width, 5 ns) was employed for the excitation light and the photodiode array was used for a detector. Transient absorption measurements on the nanosecond to microsecond time scale were conducted by the randomly interleaved pulse train (RIPT) method [37]. A picosecond laser, PL2210A (EKSPLA, 1 kHz, 25 ps, 30 μJ pulse^−1^ for 355 nm), and a supercontinuum (SC) radiation source (SC-450, Fianium, 20 MHz, pulse width: 50–100 ps depending on the wavelength, 450–2000 nm) were employed as the pump–pulse and probe sources, respectively. A 355 nm laser pulse was used to excite the samples. The measurements were performed in a benzene solution placed in a 2 mm quartz cell under stirring at room temperature. We used the mixture solution of isomer A and isomer B as was obtained by the synthesis and irradiated a 355 nm pulse laser during the measurements. By considering the duration of the measurements (usually it takes one hour) and the total photon numbers, the system probably reaches the PSS. The ratio of isomer A and isomer B at the PSS upon excitation with the 355 nm pulse is 22:78.

#### Femtosecond transient absorption measurements

Transient absorption spectra in the visible-light region were measured using a home-built setup. The overall setup was driven by a Ti:Sapphire regenerative amplifier (Spitfire, Spectra-Physics, 802 nm, 1 W, 1 kHz, 100 fs) seeded by a Ti:Sapphire oscillator (Tsunami, Spectra-Physics, 802 nm, 820 mW, 80 MHz, 100 fs). The output of the amplifier was equally divided into two portions. The first one was frequency-doubled with a 50 μm β-barium borate (BBO) crystal, and the generated second harmonics was used for excitation of the sample. The second portion was introduced into a collinear optical parametric amplifier (OPA, TOPAS-Prime, Light Conversion) and converted into the infrared pulse at 1180 nm. This 1180 nm pulse was focused into a 2 mm CaF_2_ plate after passing through a delay stage, so as to generate femtosecond white light continuum for the probe pulse. The probe pulse was divided into signal and reference pulses. The signal pulse was guided into the sample and then the both pulses were detected using a pair of multichannel photodiode array (PMA-10, Hamamatsu). The chirping of the white light continuum was evaluated by an optical Kerr effect of carbon tetrachloride and used for the corrections of the spectra. The FWHM of the cross correlation between the excitation and probe pulses was ca. 170 fs. The polarization of the excitation pulse was set to the magic angle with respect to that of the probe pulse. The typical excitation power was 100 nJ pulse^−1^ at the sample position. During the measurement, the sample solution was circulated with a home-made rotation cell with 1 mm optical length. Steady-state absorption spectra were recorded before and after the transient absorption measurement to examine photodegradation of the sample and no permanent change in absorbance was observed. We used the mixture solution of isomer A and isomer B as was obtained by the synthesis and irradiated a 400 nm pulse laser during the measurements. By considering the duration of the measurements (usually takes several hours), the system probably reaches the PSS. Under the irradiation of the 400 nm laser, the ratio of isomer A and isomer B at the PSS depends on each absorption coefficients and the efficiency for the bond cleavage. The absorption coefficients of isomer A and isomer B at 400 nm are 2.1 × 10^3^ M^−1^ cm^−1^ and 4.1 × 10^3^ M^−1^ cm^−1^, respectively.

## Supporting Information

File 1 Details of materials characterizations and analyses.
