# Supplementary material for: Excited state dynamics for visible-light sensitization of a photochromic benzil-subsituted phenoxyl-imidazolyl radical
Source: Beilstein J Org Chem. 2019 Oct 4;15:2369–79. doi: 10.3762/bjoc.15.229 (PMC6808191; doi:10.3762/bjoc.15.229)
Supplement: File 1 — Details of materials characterizations and analyses. [file Beilstein_J_Org_Chem-15-2369-s001.pdf]

BEILSTEIN JOURNAL OF ORGANIC CHEMISTRY

Supporting Information

for

Excited state dynamics for visible-light sensitization of a photochromic benzil-subsituted phenoxyl-imidazolyl radical

Yoichi Kobayashi, Yukie Mamiya, Katsuya Mutoh, Hikaru Sotome, Masafumi Koga, Hiroshi Miyasaka and Jiro Abe

Beilstein J. Org. Chem. **2019,** *15,* 2369–2379. doi:10.3762/bjoc.15.229

Details of materials characterizations and analyses

License and Terms: This is a supporting information file under the terms of the Creative Commons Attribution License (http://creativecommons.org/licenses/by/4.0). Please note that the reuse, redistribution and reproduction in particular requires that the authors and source are credited. The license is subject to the *Beilstein Journal of Organic Chemistry* terms and conditions: (https://www.beilstein-journals.org/bjoc)

Table of Contents

**1. ^{1}H NMR spectra**	**S2**
**2. HR-ESI-TOF-MS spectra**	**S4**
**3. HPLC chromatograms**	**S5**
**4. Difference in the thermal back reactions between two isomers of Benzil-PIC**	**S6**
**5. Estimation of the ratio of two isomers at the photostationary state**	**S7**
**6. Details of the SVD global analyses**	**S8**
**7. Sensitization of photochromic reaction with triplet excited states**	**S11**
**8. DFT calculations**	**S12**
**9. Reference**	**S36**

1. ^{1}H NMR spectra

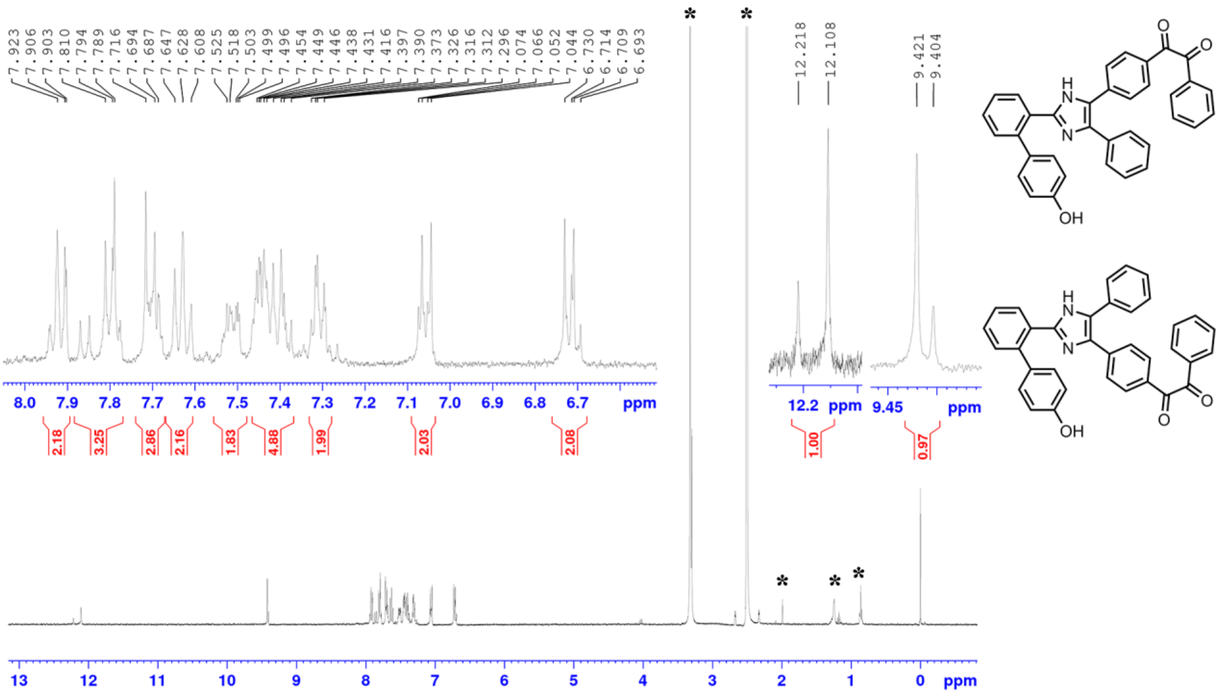

Figure S1. ^{1}H NMR spectrum of **2** in DMSO-d_6 (* solvent peaks).

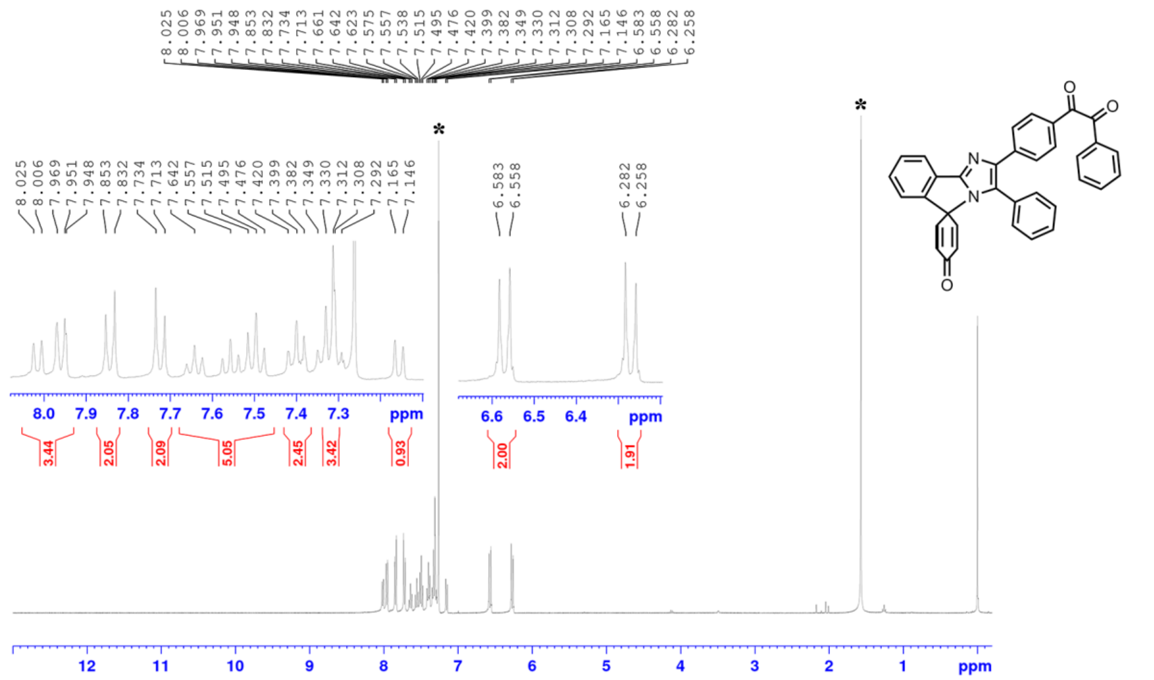

Figure S2. ^{1}H NMR spectrum of the isomer A of Benzil-PIC in $CDCl_3$ (* solvent peaks).

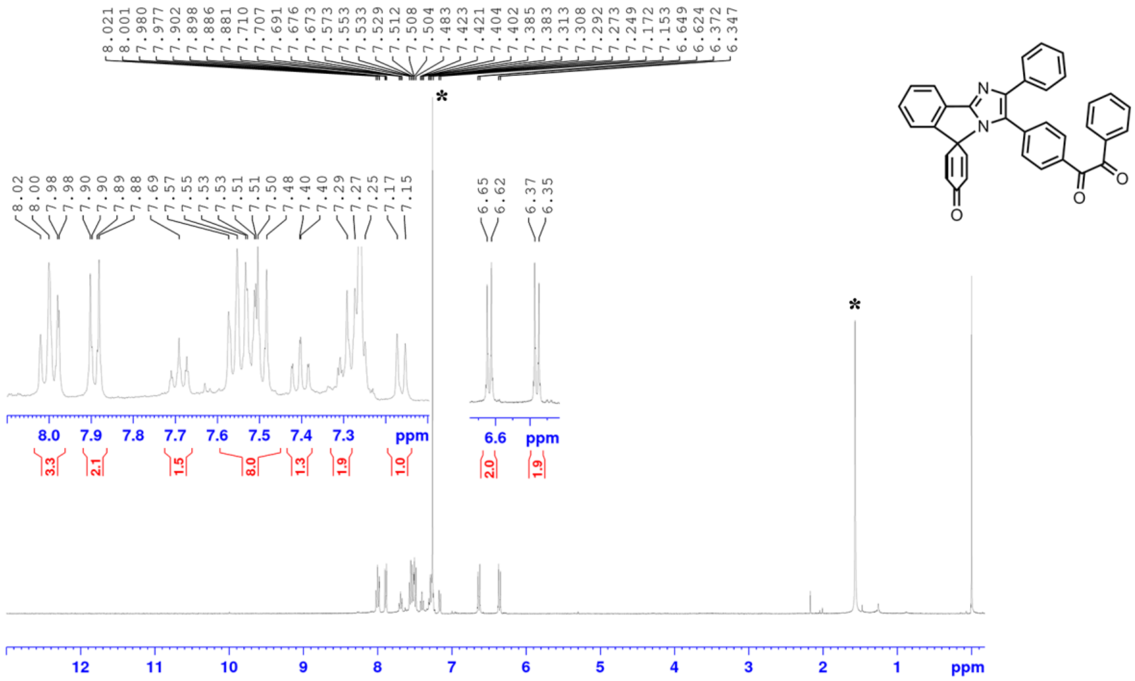

Figure S3. ^{1}H NMR spectrum of the isomer B of Benzil-PIC in $CDCl_3$ (* solvent peaks).

2. HR-ESI-TOF-MS spectra

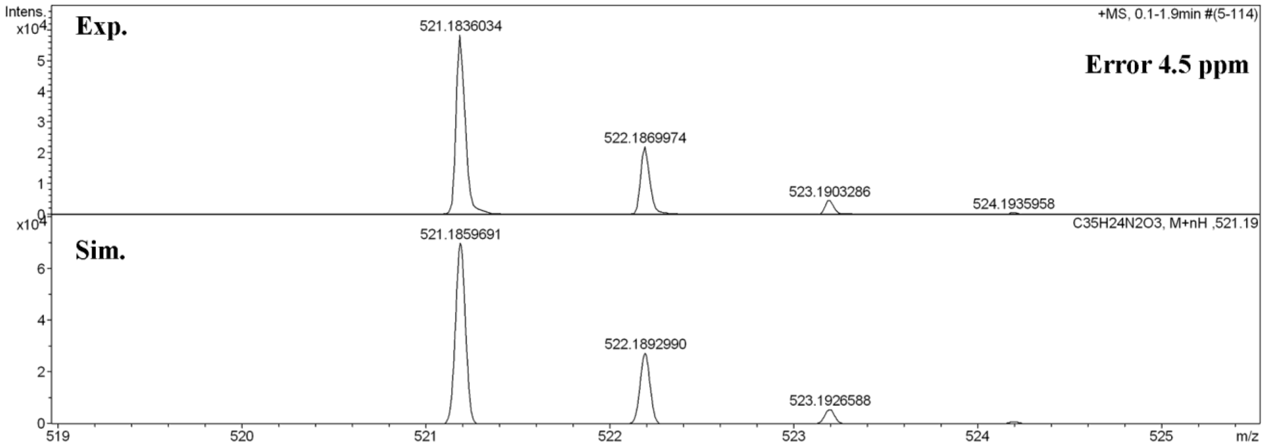

Figure S4. HR-ESI-TOF MS spectra of **2**.

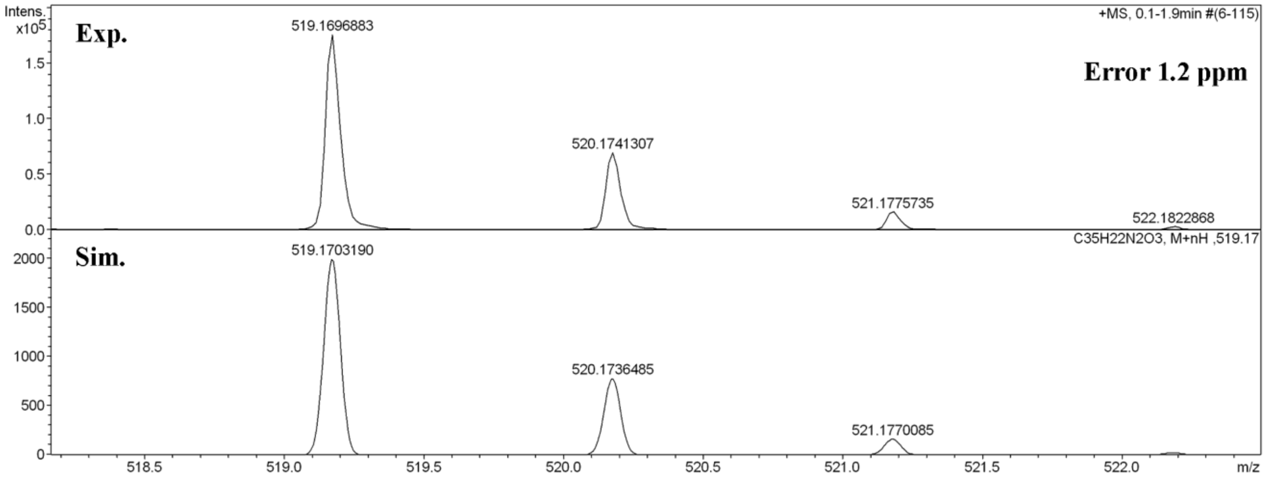

Figure S5. HR-ESI-TOF MS spectra of Benzil-PIC.

3. HPLC chromatograms

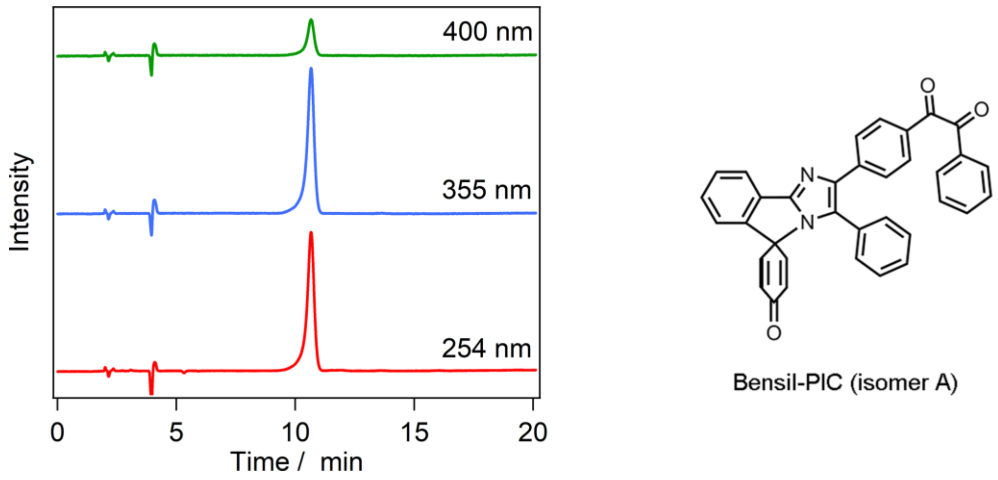

Figure S6. HPLC chromatogram of isomer A of benzil-PIC; 99% purity. HPLC analysis was performed using a reverse phase analytical column (Mightysil RP18, 25 cm × 4.6 mm, 5 µm particle) from Kanto Chemical Industries, equipped with a UV detector; the mobile phase was CH_3CN/H_3O 3:1 with a flow rate of 1.0 mL/min (detection wavelength; 254, 355, and 400 nm).

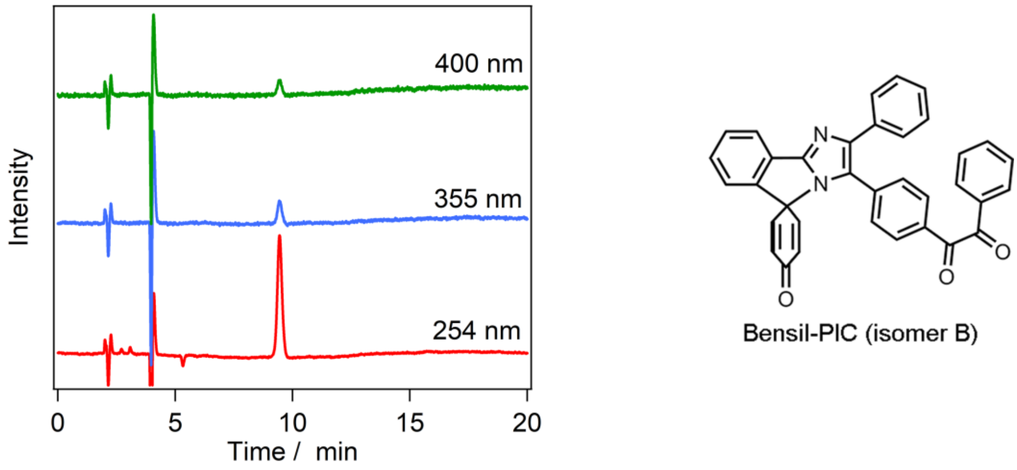

Figure S7. HPLC chromatogram of isomer B of benzil-PIC; 99% purity. HPLC analysis was performed using a reverse phase analytical column (Mightysil RP18, 25 cm × 4.6 mm, 5 µm particle) from Kanto Chemical Industries, equipped with a UV detector; the mobile phase was CH_3CN/H_3O 3:1 with a flow rate of 1.0 mL/min (detection wavelength; 254, 355, and 400 nm).

4. Difference in the thermal back reactions between two isomers of Benzil-PIC

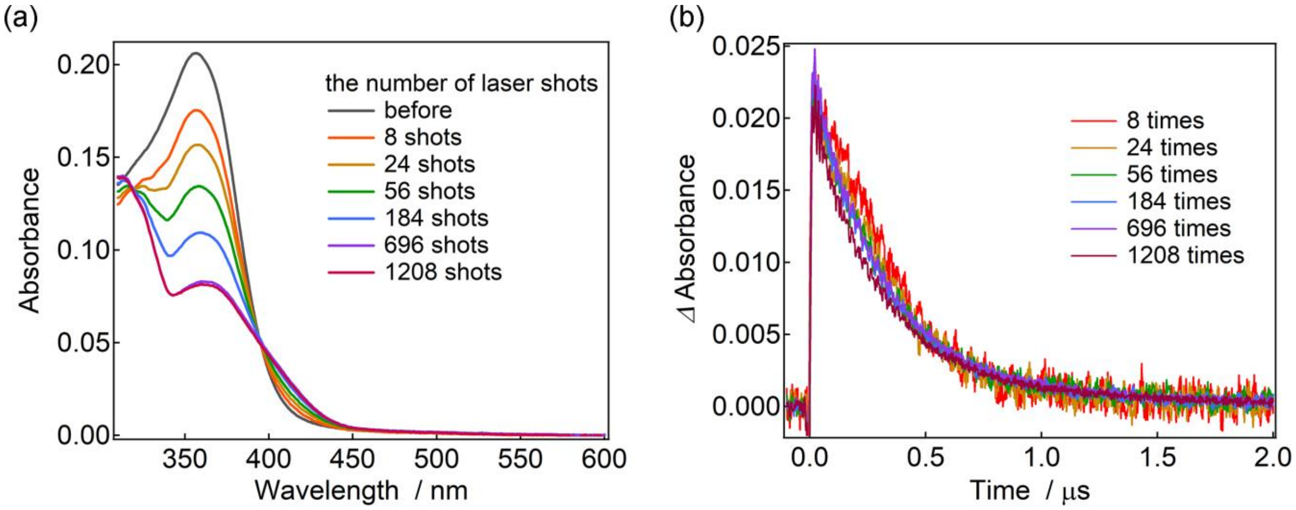

Figure S8. (a) Steady-state absorption spectra of isomer A of Benzil-PIC in benzene upon repeated irradiation of 355 nm nanosecond laser pulses (355 nm, 7 mJ pulse^{-1}). (b) Nanosecond-to-microsecond transient absorption dynamics of isomer A of Benzil-PIC in benzene at the same conditions.

5. Estimation of the ratio of two isomers at the photostationary state

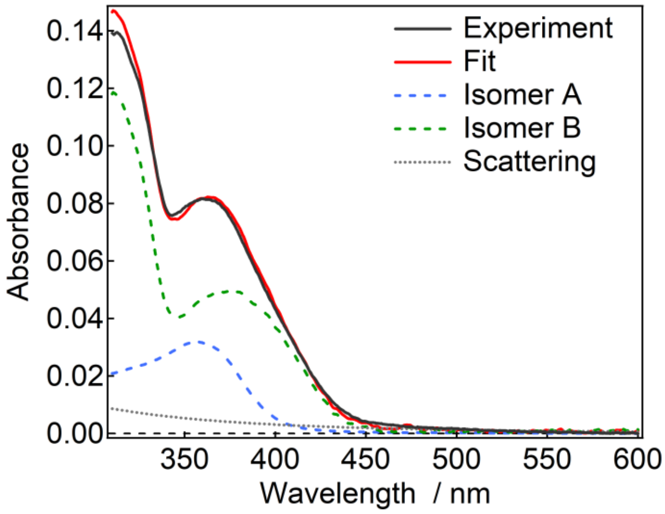

Figure S9. Absorption spectrum of Benzil-PIC at the photostationary state after the excitation with 355 nm nanosecond laser pulses. The absorption spectrum can be resolved into the two isomers by the curve fitting with pure absorption spectra of the two isomers and the small amount of the Rayleigh scattering component ($1/\lambda^4$).

6. Details of the SVD global analyses

To elucidate the details of the reaction dynamics, we performed global analyses with singular value decomposition (SVD) with the Glotaran program (http://glotaran.org). To confirm the adequacy of number of components in global fitting, the SVD was performed to the transient absorption dataset. The scree plots shown in Figure S10, which are used to determine the number of principal components, suggest that 4 or 5 components exist in benzil and Benzil-PIC although it is difficult to determine from the plots. We took the structure of the left and right singular vectors into account to determine the number of components. Left and right singular vectors represent time dependence and spectral dependence of the components. In this case, the 5th component still shows distinct structure of the left and right vectors distinct from noise in Benzil-PIC. On the other hand, the 6th component of Benzil-PIC is fluctuated (gray line in Figure S10f). Therefore, ≈5 components would be adequate to analyze the kinetics of Benzil-PIC. As compared to the number of components of Benzil-PIC, it is desirable for benzil to analyze with the fewer components than those of Benzil-PIC because benzil is used for the reference as a substructure of Benzil-PIC. Therefore, we analyzed the kinetics of benzil using 3 components. We tentatively used the three-state sequential kinetic model for benzil (Equation 1) and the five-state sequential kinetic model for Benzil-PIC (Equation 2) convolved with a Gaussian pulse.

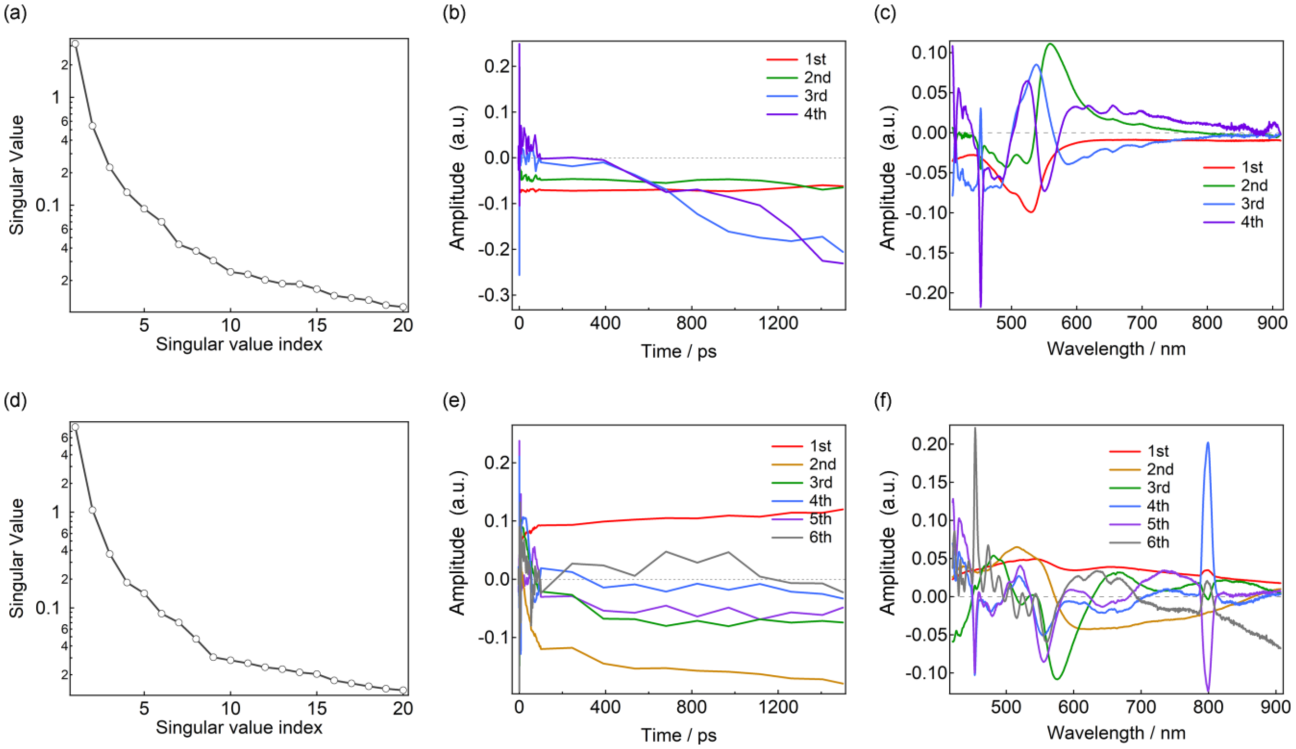

Figure S10. The singular value decomposition of the transient absorption spectra of benzil (a to c) and Benzil-PIC (d to f). (a and d) The first 20 singular values. (b and e) The first 4 and 6 left singular vectors. (c and f) The first 4 and 6 right singular vectors.

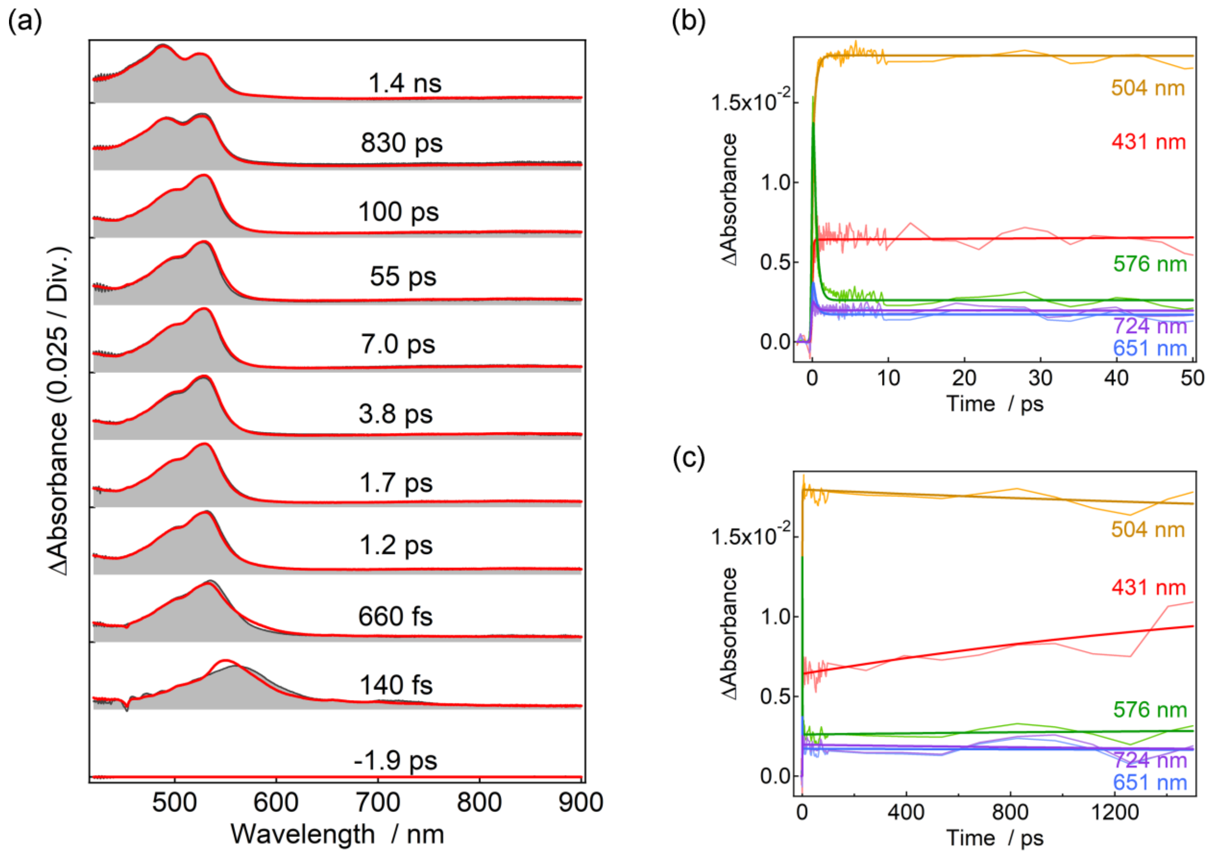

Figure S11. (a) Transient absorption spectra and fitted spectra of benzil in benzene excited at 400 nm. The red spectra show the fitted spectra by SVD global analyses assuming the three-state sequential kinetic model (b and c) Transient absorption dynamics of benzil at different time scales. Thick solid lines show the fitted dynamics.

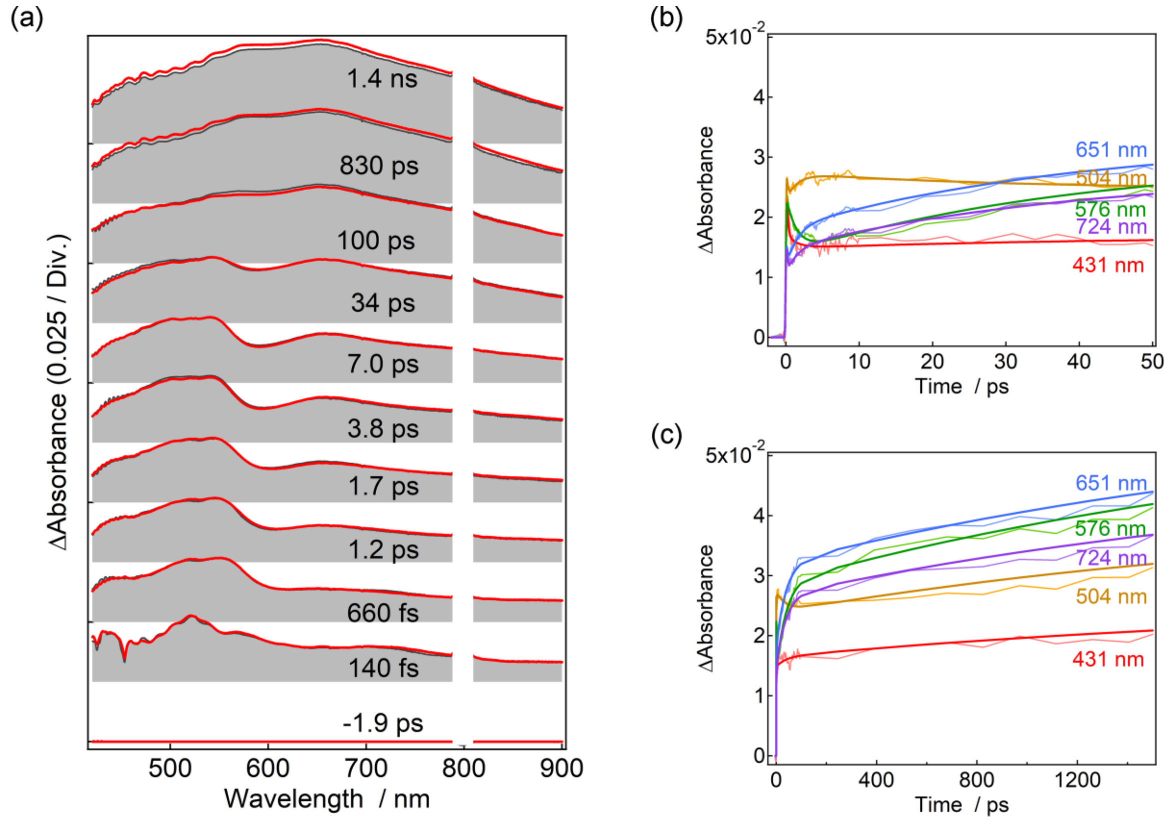

Figure S12. (a) Transient absorption spectra and fitted spectra of Benzil-PIC in benzene excited at 400 nm. The red spectra show the fitted spectra by SVD global analyses assuming the five-state sequential kinetic model (b and c) Transient absorption dynamics of Benzil-PIC at different time scales. Thick solid lines show the fitted dynamics.

7. Sensitization of photochromic reaction with triplet excited states

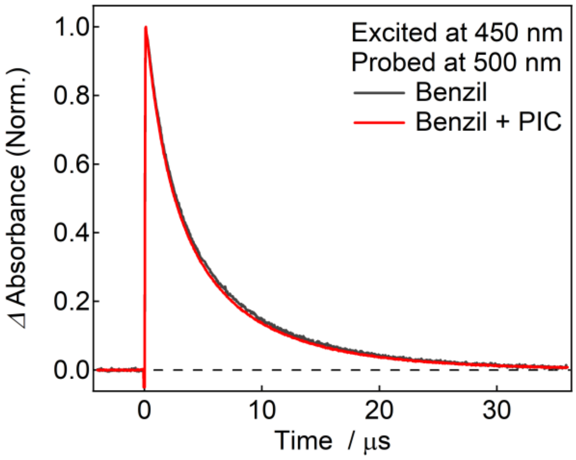

Figure S13. Microsecond transient absorption dynamics of Benzil in benzene (gray) and the mixture solution of benzil and PIC in benzene (red) excited and probed at 450 and 500 nm, respectively.

8. DFT calculations

All calculations was carried out using the Gaussian 09 program (Revision D.01).[S1] The molecular structure was fully optimized at the M05-2X/6-31+G(d,p) level of theory, and analytical second derivative was computed using vibrational analysis to confirm each stationary point to be a minimum. TDDFT calculations were performed at the MPW1PW91/6-31+G(d,p) level of the theory for the optimized structures.

Table S1. Standard orientation of the optimized geometry for the closed-ring isomer of Isomer A of Benzil-PIC.

Tag	Symbol	Coordinates (Angstroms)		
		X	Y	Z
1	C	-4.5343040	4.8339550	-0.1087920
2	C	-5.7231620	4.0997860	-0.1233790
3	C	-5.7033220	2.7045910	-0.0563070
4	C	-4.4724650	2.0768440	0.0194500
5	C	-3.2797830	2.8107210	0.0385620
6	C	-3.2960610	4.1985250	-0.0279040
7	C	-2.1871820	1.8491060	0.1070340
8	C	-4.1894340	0.5652840	0.1168770
9	C	-4.6626950	-0.1533880	-1.1167110
10	C	-5.6317340	-1.0709760	-1.0921870
11	C	-6.2792230	-1.4787900	0.1754780
12	C	-5.7762550	-0.8511180	1.4194990
13	C	-4.7935480	0.0516020	1.3959970
14	N	-2.7219810	0.5944710	0.1722450
15	C	-1.6828470	-0.3046790	0.1123530
16	C	-0.5447700	0.4952940	0.0548370
17	N	-0.8826850	1.8345120	0.0443630
18	C	0.8636860	0.0818830	-0.0027350
19	C	-1.8938520	-1.7591180	-0.0038450
20	C	-1.4321630	-2.4279980	-1.1431540
21	C	-1.6515160	-3.7935340	-1.2908720
22	C	-2.3442940	-4.5008500	-0.3092460
23	C	-2.8017400	-3.8415200	0.8294570
24	C	-2.5700520	-2.4774480	0.9865140
25	C	1.8071290	0.9681240	-0.5366310
26	C	3.1448950	0.6075630	-0.6208350
27	C	3.5624040	-0.6459460	-0.1599780
28	C	2.6260220	-1.5260940	0.3955400
29	C	1.2906410	-1.1678190	0.4745130
30	C	4.9806680	-1.0644110	-0.1899970

31	O	5.3814070	-2.1146250	0.2786780
32	C	6.0014510	-0.1688320	-0.9034530
33	C	7.2011860	0.2919990	-0.1594610
34	O	5.8090470	0.0887740	-2.0763970
35	C	8.1512290	1.0460120	-0.8571890
36	C	9.2726070	1.5298590	-0.1976690
37	C	9.4464920	1.2665020	1.1623640
38	C	8.5007700	0.5188440	1.8599610
39	C	7.3761160	0.0284030	1.2025030
40	O	-7.1791920	-2.3012420	0.1983380
41	H	-4.5764180	5.9145840	-0.1620050
42	H	-6.6712220	4.6181560	-0.1883410
43	H	-6.6224290	2.1298460	-0.0661400
44	H	-2.3711160	4.7600690	-0.0170180
45	H	-4.1781050	0.1414200	-2.0414930
46	H	-5.9793190	-1.5671800	-1.9894170
47	H	-6.2345740	-1.1833430	2.3427670
48	H	-4.4089150	0.5037080	2.3045990
49	H	-0.9010670	-1.8693560	-1.9048360
50	H	-1.2888020	-4.3034800	-2.1745190
51	H	-2.5235150	-5.5618890	-0.4296530
52	H	-3.3331810	-4.3886270	1.5981380
53	H	-2.9057290	-1.9691320	1.8813820
54	H	1.4726060	1.9354000	-0.8864100
55	H	3.8570630	1.2938830	-1.0593470
56	H	2.9695920	-2.4835590	0.7659350
57	H	0.5767650	-1.8498520	0.9176240
58	H	7.9886320	1.2379520	-1.9101330
59	H	10.0101110	2.1108210	-0.7369100
60	H	10.3213980	1.6444870	1.6771310
61	H	8.6401430	0.3135660	2.9136400
62	H	6.6512840	-0.5678540	1.7402340

SCF Done: E(RM052X) = -1682.07194943

Zero-point correction = 0.484395 (Hartree/Particle)
Thermal correction to Energy = 0.514380
Thermal correction to Enthalpy = 0.515324
Thermal correction to Gibbs Free Energy = 0.419921

Sum of electronic and zero-point Energies = -1681.587555
Sum of electronic and thermal Energies = -1681.557569
Sum of electronic and thermal Enthalpies = -1681.556625
Sum of electronic and thermal Free Energies = -1681.652029

Low frequencies --- -15.6158 -7.3907 -4.7849 -0.0025 -0.0020 -0.0015
Low frequencies --- 4.2114 17.0347 19.5463

The Result for the TDDFT calculation of Isomer A of Benzil-PIC

Excited State 1: Singlet-A 2.9259 eV 423.75 nm f=0.0004 <S**2>=0.000
135 ->136 0.70311
This state for optimization and/or second-order correction.
Total Energy, E(TD-HF/TD-KS) = -1681.71934799
Copying the excited state density for this state as the 1-particle RhoCI density.

Excited State 2: Singlet-A 2.9476 eV 420.63 nm f=0.0019 <S**2>=0.000
134 ->137 0.67289

Excited State 3: Singlet-A 3.3545 eV 369.60 nm f=0.4650 <S**2>=0.000
135 ->137 0.69239

Excited State 4: Singlet-A 3.3566 eV 369.38 nm f=0.0009 <S**2>=0.000
127 ->136 -0.31432
128 ->136 0.61322

Excited State 5: Singlet-A 3.7322 eV 332.20 nm f=0.0367 <S**2>=0.000
121 ->137 0.16761
134 ->138 0.60262
134 ->141 0.11111
135 ->138 0.24962

Excited State 6: Singlet-A 3.9208 eV 316.23 nm f=0.0659 <S**2>=0.000
134 ->138 -0.19248
135 ->138 0.53794
135 ->139 -0.37383

Excited State 7: Singlet-A 3.9311 eV 315.39 nm f=0.3054 <S**2>=0.000
134 ->138 -0.12780
135 ->138 0.35971
135 ->139 0.57393
135 ->140 -0.10136

Excited State 8: Singlet-A 4.0772 eV 304.09 nm f=0.0015 <S**2>=0.000
134 ->136 0.69654

Excited State 9: Singlet-A 4.2382 eV 292.54 nm f=0.1177 <S**2>=0.000

126 ->139 -0.11429

133 ->137 -0.11417

135 ->139 0.11912

135 ->140 0.64432

Excited State 10: Singlet-A 4.2940 eV 288.74 nm f=0.0178 <S**2>=0.000

127 ->137 -0.10703

132 ->137 -0.27994

133 ->136 0.25554

133 ->137 0.47623

135 ->140 0.16842

135 ->142 0.14419

135 ->145 0.11100

Excited State 11: Singlet-A 4.3010 eV 288.27 nm f=0.0036 <S**2>=0.000

129 ->136 -0.12741

130 ->136 0.28202

132 ->136 0.32103

132 ->137 0.12595

133 ->136 0.43117

133 ->137 -0.22750

Excited State 12: Singlet-A 4.3666 eV 283.94 nm f=0.0064 <S**2>=0.000

126 ->136 0.20343

127 ->136 -0.30682

128 ->136 -0.18191

130 ->136 0.37678

132 ->136 0.21393

133 ->136 -0.35424

Excited State 13: Singlet-A 4.3972 eV 281.96 nm f=0.0022 <S**2>=0.000

126 ->136 -0.30892

129 ->136 0.45942

130 ->136 -0.15666

132 ->136 0.38259

Excited State 14: Singlet-A 4.4604 eV 277.97 nm f=0.0534 <S**2>=0.000

129 ->137 -0.18748

130 ->137 -0.19753

131 ->137 0.49445
131 ->138 0.13800
132 ->137 0.27604
133 ->137 0.19022

Excited State 15: Singlet-A 4.5055 eV 275.18 nm f=0.0227 <S**2>=0.000
126 ->136 0.53240
128 ->136 -0.11476
129 ->136 0.28749
130 ->136 -0.20491
133 ->136 0.16646
133 ->137 -0.10306

Excited State 16: Singlet-A 4.5102 eV 274.90 nm f=0.1750 <S**2>=0.000
126 ->136 0.16365
131 ->137 -0.37615
132 ->137 0.38534
133 ->137 0.25669
135 ->141 -0.25771

Excited State 17: Singlet-A 4.5650 eV 271.60 nm f=0.0120 <S**2>=0.000
132 ->137 0.11982
133 ->137 -0.12036
135 ->141 -0.10794
135 ->142 0.63092

Excited State 18: Singlet-A 4.5948 eV 269.83 nm f=0.0450 <S**2>=0.000
127 ->137 -0.10556
129 ->137 -0.12119
130 ->137 -0.18831
131 ->137 -0.17716
132 ->137 0.17914
135 ->141 0.54786

Excited State 19: Singlet-A 4.6289 eV 267.85 nm f=0.0006 <S**2>=0.000
126 ->136 0.17369
127 ->136 0.39423
128 ->136 0.18066
129 ->136 -0.15768

130 ->136 -0.14840
132 ->136 0.36624
133 ->136 -0.24380
135 ->141 0.14842

Excited State 20: Singlet-A 4.7094 eV 263.27 nm f=0.0329 <S**2>=0.000
127 ->137 0.24300
128 ->137 0.11313
129 ->137 0.30709
130 ->137 0.32937
131 ->137 0.18462
131 ->138 -0.10335
132 ->137 0.19268
133 ->137 0.19787
135 ->141 0.20342
135 ->142 0.10227

Excited State 21: Singlet-A 4.8499 eV 255.64 nm f=0.0087 <S**2>=0.000
127 ->137 -0.17793
128 ->137 -0.12202
129 ->137 0.54239
129 ->138 -0.12773
130 ->137 -0.30621

Excited State 22: Singlet-A 4.8600 eV 255.11 nm f=0.0002 <S**2>=0.000
131 ->136 0.70119

Excited State 23: Singlet-A 4.8641 eV 254.90 nm f=0.0102 <S**2>=0.000
127 ->137 -0.25149
127 ->138 0.12943
128 ->137 -0.14352
130 ->137 0.34893
132 ->137 0.18302
132 ->138 0.11354
133 ->137 -0.10872
133 ->138 -0.29273
135 ->145 0.28092

Excited State 24: Singlet-A 4.9117 eV 252.43 nm f=0.0574 <S**2>=0.000

121 ->137 -0.12296
127 ->137 0.35331
128 ->137 0.21273
130 ->137 -0.19630
132 ->138 0.25402
133 ->138 -0.32620
135 ->145 0.21308

Excited State 25: Singlet-A 4.9299 eV 251.49 nm f=0.0014 <S**2>=0.000
124 ->136 0.18117
125 ->136 -0.12341
127 ->136 0.29656
128 ->136 0.16714
129 ->136 0.36786
130 ->136 0.38104
132 ->136 -0.18410

Excited State 26: Singlet-A 4.9673 eV 249.60 nm f=0.0066 <S**2>=0.000
126 ->139 0.14492
135 ->144 0.63484

Excited State 27: Singlet-A 4.9900 eV 248.46 nm f=0.0026 <S**2>=0.000
134 ->139 0.68982

Excited State 28: Singlet-A 5.0255 eV 246.71 nm f=0.0051 <S**2>=0.000
121 ->137 0.11648
122 ->136 -0.10240
124 ->136 0.46768
125 ->136 -0.39911
127 ->136 -0.14649
129 ->136 -0.10884
130 ->136 -0.14101

Excited State 29: Singlet-A 5.0376 eV 246.12 nm f=0.0053 <S**2>=0.000
121 ->137 0.50752
122 ->137 0.19741
124 ->136 -0.11066
128 ->137 0.10803
131 ->138 0.16916

132 ->137 0.12221
134 ->138 -0.20173

Excited State 30: Singlet-A 5.0903 eV 243.57 nm f=0.0543 <S**2>=0.000
121 ->137 -0.10160
127 ->137 0.10671
129 ->138 -0.13754
130 ->137 0.10434
130 ->138 -0.17503
131 ->137 -0.12435
131 ->138 0.47643
132 ->138 0.22320
133 ->138 0.20685

Excited State 31: Singlet-A 5.1093 eV 242.66 nm f=0.0009 <S**2>=0.000
127 ->137 -0.31187
128 ->137 0.58459
131 ->138 0.10720

Excited State 32: Singlet-A 5.1352 eV 241.44 nm f=0.0646 <S**2>=0.000
128 ->137 0.11443
131 ->138 -0.15104
133 ->138 0.37550
133 ->139 -0.15843
135 ->143 0.21834
135 ->145 0.39924
135 ->146 0.10237

Excited State 33: Singlet-A 5.1828 eV 239.22 nm f=0.0404 <S**2>=0.000
126 ->137 0.36871
127 ->137 -0.16848
131 ->138 -0.12848
132 ->138 0.34148
132 ->139 -0.13525
133 ->138 0.11974
135 ->144 0.10014
135 ->145 -0.14110
135 ->146 -0.22046

Excited State 34: Singlet-A 5.1969 eV 238.57 nm f=0.0138 <S**2>=0.000

126 ->137 0.53132

132 ->138 -0.22924

135 ->145 0.16972

135 ->146 0.27113

Excited State 35: Singlet-A 5.2307 eV 237.03 nm f=0.0017 <S**2>=0.000

125 ->136 -0.10481

132 ->138 -0.18689

133 ->139 -0.27755

134 ->143 0.11707

135 ->143 0.38883

135 ->146 -0.33684

Excited State 36: Singlet-A 5.2499 eV 236.16 nm f=0.0470 <S**2>=0.000

128 ->139 0.12519

132 ->139 0.24311

133 ->138 0.13241

133 ->139 0.48248

135 ->143 0.25774

135 ->146 -0.20751

Excited State 37: Singlet-A 5.2607 eV 235.68 nm f=0.0351 <S**2>=0.000

132 ->138 0.21132

133 ->138 -0.10580

135 ->143 0.40958

135 ->145 -0.22805

135 ->146 0.39974

Excited State 38: Singlet-A 5.2742 eV 235.08 nm f=0.0344 <S**2>=0.000

123 ->136 0.18925

124 ->136 0.30554

125 ->136 0.45356

129 ->139 0.17958

130 ->139 -0.16726

132 ->138 -0.14374

Excited State 39: Singlet-A 5.3070 eV 233.62 nm f=0.0055 <S**2>=0.000

125 ->136 0.12107

126 ->139 0.23048

132 ->140 -0.13471

133 ->140 -0.11711

134 ->140 0.53356

134 ->142 -0.10516

135 ->144 -0.10384

Excited State 40: Singlet-A 5.3297 eV 232.63 nm f=0.0281 <S**2>=0.000

128 ->139 0.32059

128 ->140 -0.15109

129 ->138 0.19247

130 ->138 0.18280

130 ->139 -0.15970

131 ->138 0.11899

132 ->139 -0.25578

134 ->140 0.15082

135 ->145 0.10020

133 ->138 0.22100

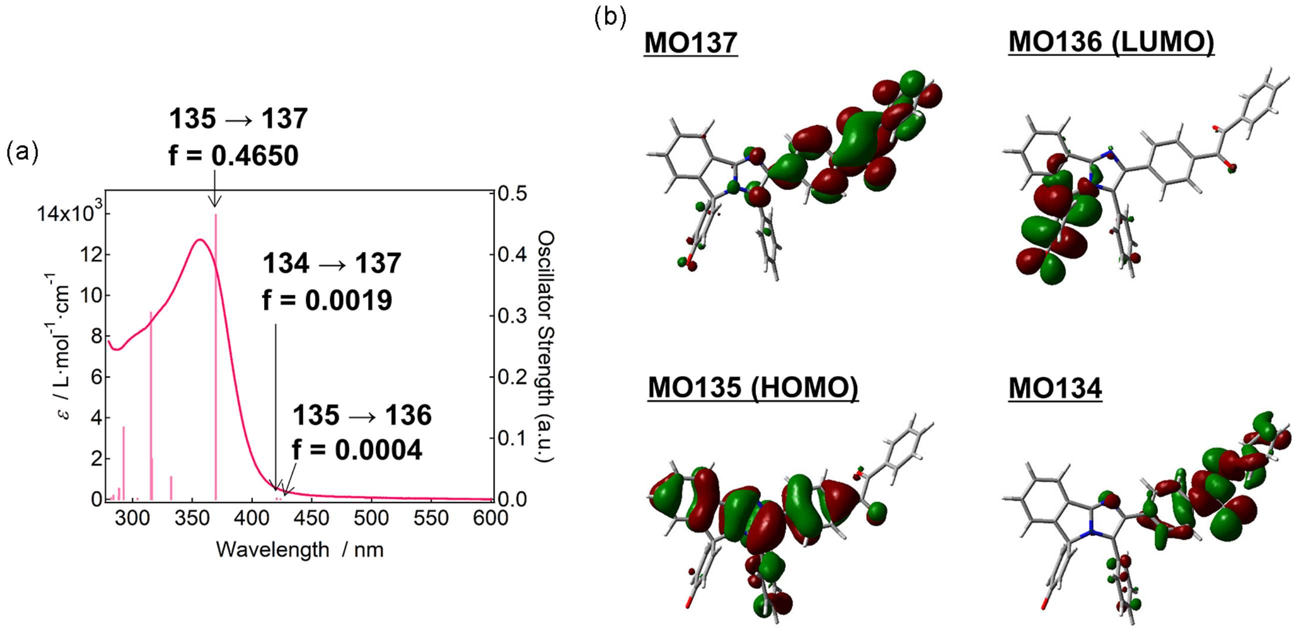

Figure S14. (a) UV–vis absorption spectrum of the closed-ring isomer of isomer A of Benzil-PIC in benzene. The calculated spectrum (MPW1PW91/6-31+G(d,p)//M05-2X/6-31+G(d,p) level of the theory) is shown by the vertical lines. (b) The relevant molecular orbitals of isomer A of Benzil-PIC calculated at the M05-2X/6-31+G(d,p) level of the theory.

Table S2. Standard orientation of the optimized geometry for the closed-ring isomer of isomer B of Benzil-PIC.

Tag	Symbol	Coordinates (Angstroms)		
		X	Y	Z
1	C	-7.2911450	-0.0734050	0.5917970
2	C	-6.9953580	-1.4392900	0.5947190
3	C	-5.6885720	-1.8887120	0.3916340
4	C	-4.7008270	-0.9398680	0.1947450
5	C	-4.9946510	0.4284170	0.1886290
6	C	-6.2937110	0.8792730	0.3891000
7	C	-3.7376390	1.1338130	-0.0187760
8	C	-3.2004740	-1.1793270	-0.0657180
9	C	-3.0762250	-1.9264700	-1.3667960
10	C	-2.6118700	-3.1757120	-1.4392430
11	C	-2.0954660	-3.8785860	-0.2409580
12	C	-2.0751500	-3.1152180	1.0283060
13	C	-2.5568550	-1.8725980	1.1031740
14	N	-2.7428000	0.2132520	-0.1766710
15	C	-1.5497600	0.9060640	-0.2274900

16	C	-1.9266470	2.2443190	-0.1473590
17	N	-3.2928230	2.3633260	-0.0094020
18	C	-1.0734110	3.4431170	-0.2044330
19	C	-0.2283030	0.2616310	-0.2335760
20	C	0.7209930	0.6467540	0.7211820
21	C	1.9738950	0.0492960	0.7498830
22	C	2.2903370	-0.9528200	-0.1738610
23	C	1.3470460	-1.3389590	-1.1316680
24	C	0.1002790	-0.7329290	-1.1646350
25	C	-1.4831080	4.6023200	0.4636110
26	C	-0.6998760	5.7513550	0.4242460
27	C	0.5008660	5.7586210	-0.2832960
28	C	0.9075550	4.6114700	-0.9620090
29	C	0.1237920	3.4622590	-0.9291660
30	O	-1.6962730	-5.0286230	-0.3022650
31	C	3.6253000	-1.6008130	-0.2171430
32	O	3.9269000	-2.4414760	-1.0423170
33	C	4.6597650	-1.2389270	0.8558790
34	O	4.3465550	-1.3950990	2.0206440
35	C	6.0017250	-0.7670200	0.4322890
36	C	6.3257740	-0.5528020	-0.9111830
37	C	7.5873380	-0.0700480	-1.2468990
38	C	8.5208250	0.1971960	-0.2484160
39	C	8.1982090	-0.0162120	1.0930000
40	C	6.9409860	-0.4953700	1.4336870
41	H	-8.3122390	0.2494520	0.7509040
42	H	-7.7883330	-2.1580920	0.7569020
43	H	-5.4534820	-2.9470220	0.3913090
44	H	-6.5138950	1.9387700	0.3874760
45	H	-3.4474940	-1.4007690	-2.2408480
46	H	-2.5737710	-3.7259210	-2.3712520
47	H	-1.6391290	-3.6172950	1.8826690
48	H	-2.5385570	-1.3073970	2.0290100
49	H	0.4664600	1.4163050	1.4392320
50	H	2.6907620	0.3451010	1.5035630
51	H	1.6149220	-2.1078820	-1.8453710
52	H	-0.6175500	-1.0165520	-1.9224520
53	H	-2.4204080	4.5879840	1.0047200
54	H	-1.0270510	6.6418600	0.9469150

55	H	1.1104300	6.6533330	-0.3129070
56	H	1.8306700	4.6139440	-1.5285040
57	H	0.4347300	2.5829710	-1.4796620
58	H	5.6085020	-0.7771490	-1.6889680
59	H	7.8417920	0.0927970	-2.2863820
60	H	9.5019520	0.5712090	-0.5142400
61	H	8.9265610	0.1919170	1.8665100
62	H	6.6631990	-0.6669360	2.4658600

SCF Done: E(RM052X) = -1682.06936648

Zero-point correction = 0.484362 (Hartree/Particle)
Thermal correction to Energy = 0.515169
Thermal correction to Enthalpy = 0.516114
Thermal correction to Gibbs Free Energy = 0.417471
Sum of electronic and zero-point Energies = -1681.585004
Sum of electronic and thermal Energies = -1681.554197
Sum of electronic and thermal Enthalpies = -1681.553253
Sum of electronic and thermal Free Energies = -1681.651895

Low frequencies --- -4.3160 -1.9542 -0.0022 -0.0021 -0.0008 2.7981
Low frequencies --- 9.0325 15.7886 20.1891

The Result for the TDDFT calculation of Isomer B of Benzil-PIC

Excited State 1: Singlet-A 2.8390 eV 436.72 nm f=0.0510 <S**2>=0.000
135 ->136 0.59567
135 ->137 -0.37415
This state for optimization and/or second-order correction.
Total Energy, E(TD-HF/TD-KS) = -1681.72018008
Copying the excited state density for this state as the 1-particle RhoCI density.

Excited State 2: Singlet-A 2.9139 eV 425.50 nm f=0.0005 <S**2>=0.000
134 ->136 0.61091
134 ->137 0.29730

Excited State 3: Singlet-A 3.0151 eV 411.21 nm f=0.1352 <S**2>=0.000
135 ->136 0.36705
135 ->137 0.59089
135 ->138 -0.10602

Excited State 4: Singlet-A 3.3406 eV 371.15 nm f=0.0000 <S**2>=0.000
131 ->136 -0.35930
131 ->137 0.58641

Excited State 5: Singlet-A 3.6321 eV 341.36 nm f=0.0668 <S**2>=0.000
134 ->138 0.10961
135 ->138 0.67919

Excited State 6: Singlet-A 3.7531 eV 330.35 nm f=0.0002 <S**2>=0.000
120 ->136 -0.13517
121 ->136 -0.10047
134 ->136 -0.13186
134 ->137 0.15289
134 ->138 0.60232
134 ->139 0.11434
135 ->138 -0.12011

Excited State 7: Singlet-A 3.9652 eV 312.68 nm f=0.1856 <S**2>=0.000
135 ->139 0.67109
135 ->140 0.17159

Excited State 8: Singlet-A 4.1273 eV 300.40 nm f=0.0011 <S**2>=0.000
133 ->136 0.64944
135 ->140 -0.17365

Excited State 9: Singlet-A 4.1565 eV 298.29 nm f=0.0162 <S**2>=0.000
132 ->136 0.31656
134 ->136 -0.25569
134 ->137 0.51977
134 ->138 -0.13358

Excited State 10: Singlet-A 4.1697 eV 297.34 nm f=0.2147 <S**2>=0.000
132 ->136 -0.12126
133 ->136 0.16126
135 ->139 -0.17387
135 ->140 0.63007

Excited State 11: Singlet-A 4.1901 eV 295.90 nm f=0.1010 <S**2>=0.000
132 ->136 0.53040
132 ->137 -0.10029
133 ->136 0.11233
134 ->136 0.11975
134 ->137 -0.31070
134 ->138 0.12016
135 ->140 0.11875
135 ->141 -0.10282

Excited State 12: Singlet-A 4.3070 eV 287.87 nm f=0.0288 <S**2>=0.000
127 ->136 -0.15075
128 ->137 0.10459
129 ->136 0.21410
132 ->137 0.46372
133 ->137 0.33595
135 ->141 -0.20622

Excited State 13: Singlet-A 4.3575 eV 284.53 nm f=0.0339 <S**2>=0.000
126 ->136 0.15027
127 ->136 0.18269
130 ->136 -0.15818
132 ->136 0.22852

133 ->136 -0.11694
133 ->137 0.36498
135 ->141 0.40636

Excited State 14: Singlet-A 4.3653 eV 284.02 nm f=0.0077 <S**2>=0.000
128 ->136 -0.10727
129 ->136 -0.23630
130 ->136 0.52495
130 ->137 0.22804
130 ->138 0.11679
133 ->137 0.19844

Excited State 15: Singlet-A 4.3917 eV 282.31 nm f=0.0449 <S**2>=0.000
127 ->136 -0.14918
128 ->136 0.21434
128 ->137 -0.27443
129 ->136 -0.20402
129 ->137 0.18329
130 ->136 -0.12206
132 ->137 -0.26510
133 ->137 0.31902
135 ->141 -0.24928

Excited State 16: Singlet-A 4.4173 eV 280.68 nm f=0.0135 <S**2>=0.000
127 ->136 0.10549
127 ->137 -0.12204
128 ->136 0.26904
128 ->137 -0.30900
129 ->136 -0.14192
129 ->137 0.23454
132 ->137 0.36487
133 ->137 -0.27187

Excited State 17: Singlet-A 4.4568 eV 278.19 nm f=0.1076 <S**2>=0.000
128 ->136 0.27798
129 ->136 0.45692
129 ->137 0.15937
130 ->136 0.28285
132 ->136 -0.11839

132 ->137 -0.17034

Excited State 18: Singlet-A 4.5165 eV 274.52 nm f=0.0142 <S**2>=0.000
126 ->136 0.18872
127 ->136 0.49605
135 ->141 -0.34116
135 ->142 -0.22136

Excited State 19: Singlet-A 4.5900 eV 270.12 nm f=0.0007 <S**2>=0.000
126 ->136 -0.10270
131 ->136 0.58438
131 ->137 0.34167
131 ->138 -0.10560

Excited State 20: Singlet-A 4.6058 eV 269.19 nm f=0.0267 <S**2>=0.000
126 ->136 0.48298
126 ->137 -0.14209
127 ->136 -0.21088
127 ->137 0.13911
128 ->137 0.18976
129 ->136 -0.11339
129 ->137 0.22575
130 ->136 0.10984
131 ->137 0.10148
135 ->141 -0.11060

Excited State 21: Singlet-A 4.6607 eV 266.02 nm f=0.1442 <S**2>=0.000
126 ->136 0.13067
126 ->137 0.18720
127 ->137 -0.28488
128 ->136 0.14681
129 ->137 -0.25884
135 ->141 -0.15148
135 ->142 0.44732
135 ->143 0.12532

Excited State 22: Singlet-A 4.7023 eV 263.67 nm f=0.0164 <S**2>=0.000
126 ->137 0.39630
127 ->137 0.48254

128 ->136 0.12999

129 ->137 -0.19765

Excited State 23: Singlet-A 4.7267 eV 262.31 nm f=0.0437 <S**2>=0.000

126 ->136 -0.13307

127 ->136 0.13399

127 ->137 0.27608

128 ->136 -0.20951

128 ->137 -0.10195

129 ->136 0.13251

129 ->137 0.25109

133 ->138 0.16089

135 ->142 0.39558

Excited State 24: Singlet-A 4.8270 eV 256.86 nm f=0.0195 <S**2>=0.000

126 ->136 -0.20090

126 ->137 -0.16553

127 ->136 0.12582

127 ->137 0.11500

128 ->136 0.36659

128 ->137 0.32811

128 ->138 -0.10127

129 ->136 -0.15495

133 ->138 0.25654

135 ->144 0.11290

Excited State 25: Singlet-A 4.8436 eV 255.97 nm f=0.0188 <S**2>=0.000

127 ->136 -0.12616

128 ->136 -0.10192

128 ->137 -0.11060

133 ->138 0.55737

133 ->142 -0.10712

135 ->145 -0.16769

135 ->146 0.14345

Excited State 26: Singlet-A 4.9080 eV 252.61 nm f=0.0189 <S**2>=0.000

129 ->138 -0.11723

130 ->136 -0.27318

130 ->137 0.52042

130 ->138 0.25265
132 ->138 -0.15261

Excited State 27: Singlet-A 4.9346 eV 251.25 nm f=0.0054 <S**2>=0.000
126 ->138 -0.10109
130 ->137 0.13724
132 ->138 0.51925
135 ->144 -0.37240

Excited State 28: Singlet-A 4.9569 eV 250.12 nm f=0.0221 <S**2>=0.000
124 ->136 -0.14875
124 ->137 0.13653
125 ->136 0.14341
132 ->138 0.28994
135 ->144 0.42355
135 ->145 -0.22490
135 ->146 0.11146

Excited State 29: Singlet-A 4.9700 eV 249.46 nm f=0.0528 <S**2>=0.000
120 ->136 -0.24402
120 ->137 -0.11533
121 ->136 -0.17627
126 ->136 0.10070
126 ->137 -0.11474
128 ->137 -0.12449
129 ->136 0.10248
132 ->138 0.18478
133 ->138 0.14265
133 ->142 0.13346
134 ->138 -0.14576
135 ->144 0.20566
135 ->145 0.28507
135 ->146 -0.17445

Excited State 30: Singlet-A 4.9843 eV 248.75 nm f=0.0070 <S**2>=0.000
124 ->136 -0.17469
124 ->137 0.20396
125 ->136 0.24444
125 ->137 -0.19594

126 ->137 0.23062

128 ->137 0.15177

129 ->136 -0.13534

129 ->137 0.18628

130 ->137 0.13031

133 ->138 0.12424

133 ->142 0.10161

135 ->145 0.24172

135 ->146 -0.13241

Excited State 31: Singlet-A 5.0152 eV 247.22 nm f=0.0138 <S**2>=0.000

120 ->136 0.27557

120 ->137 0.12624

121 ->136 0.21868

121 ->137 0.11544

124 ->136 0.11118

124 ->137 -0.11219

125 ->136 -0.17360

125 ->137 0.17608

126 ->137 0.11061

132 ->138 0.10282

133 ->138 0.15429

134 ->138 0.14948

135 ->144 0.18228

135 ->145 0.16897

135 ->146 -0.11285

Excited State 32: Singlet-A 5.0190 eV 247.03 nm f=0.0013 <S**2>=0.000

120 ->136 -0.16148

121 ->136 -0.13769

124 ->136 0.10907

124 ->137 -0.13717

125 ->136 -0.25134

125 ->137 0.17217

126 ->136 -0.10825

126 ->137 0.25337

128 ->136 -0.10279

128 ->137 0.16522

129 ->137 0.18154

130 ->137 0.24172
130 ->138 -0.23198

Excited State 33: Singlet-A 5.0599 eV 245.03 nm f=0.0124 <S**2>=0.000
135 ->142 -0.15365
135 ->143 0.65899

Excited State 34: Singlet-A 5.0886 eV 243.65 nm f=0.0143 <S**2>=0.000
126 ->137 0.23768
127 ->137 -0.10148
128 ->137 0.18699
128 ->138 -0.11345
129 ->137 0.21261
129 ->138 -0.20637
130 ->137 -0.21910
130 ->138 0.42716

Excited State 35: Singlet-A 5.1578 eV 240.38 nm f=0.0151 <S**2>=0.000
124 ->136 0.26634
124 ->137 -0.17517
125 ->136 0.47589
125 ->137 0.19048
125 ->138 -0.14507
127 ->138 0.17939
132 ->139 -0.11001

Excited State 36: Singlet-A 5.1806 eV 239.32 nm f=0.0186 <S**2>=0.000
126 ->138 0.11737
127 ->138 0.19805
129 ->138 -0.11572
133 ->139 0.10712
135 ->145 0.32478
135 ->146 0.47820

Excited State 37: Singlet-A 5.2077 eV 238.08 nm f=0.0683 <S**2>=0.000
126 ->138 0.18327
127 ->138 0.39486
128 ->138 -0.12214
129 ->138 -0.17103

130 ->138 -0.11275

134 ->139 -0.21220

135 ->145 -0.26001

135 ->146 -0.19330

Excited State 38: Singlet-A 5.2318 eV 236.98 nm f=0.0045 <S**2>=0.000

124 ->136 -0.13366

124 ->137 0.10193

125 ->136 -0.12457

125 ->137 0.10040

127 ->138 0.20528

129 ->138 0.23288

131 ->138 0.18978

134 ->139 0.46421

134 ->141 -0.10496

Excited State 39: Singlet-A 5.2404 eV 236.59 nm f=0.0057 <S**2>=0.000

124 ->136 0.15819

125 ->137 -0.22260

127 ->138 -0.16217

129 ->138 -0.16008

131 ->138 0.39639

131 ->139 -0.25050

131 ->140 -0.17316

132 ->139 -0.14452

133 ->139 -0.18348

Excited State 40: Singlet-A 5.2538 eV 235.99 nm f=0.0083 <S**2>=0.000

124 ->136 0.16756

125 ->137 -0.24111

126 ->138 0.13569

129 ->138 -0.21236

131 ->138 -0.31070

131 ->139 0.15628

131 ->140 0.11376

134 ->139 0.34806

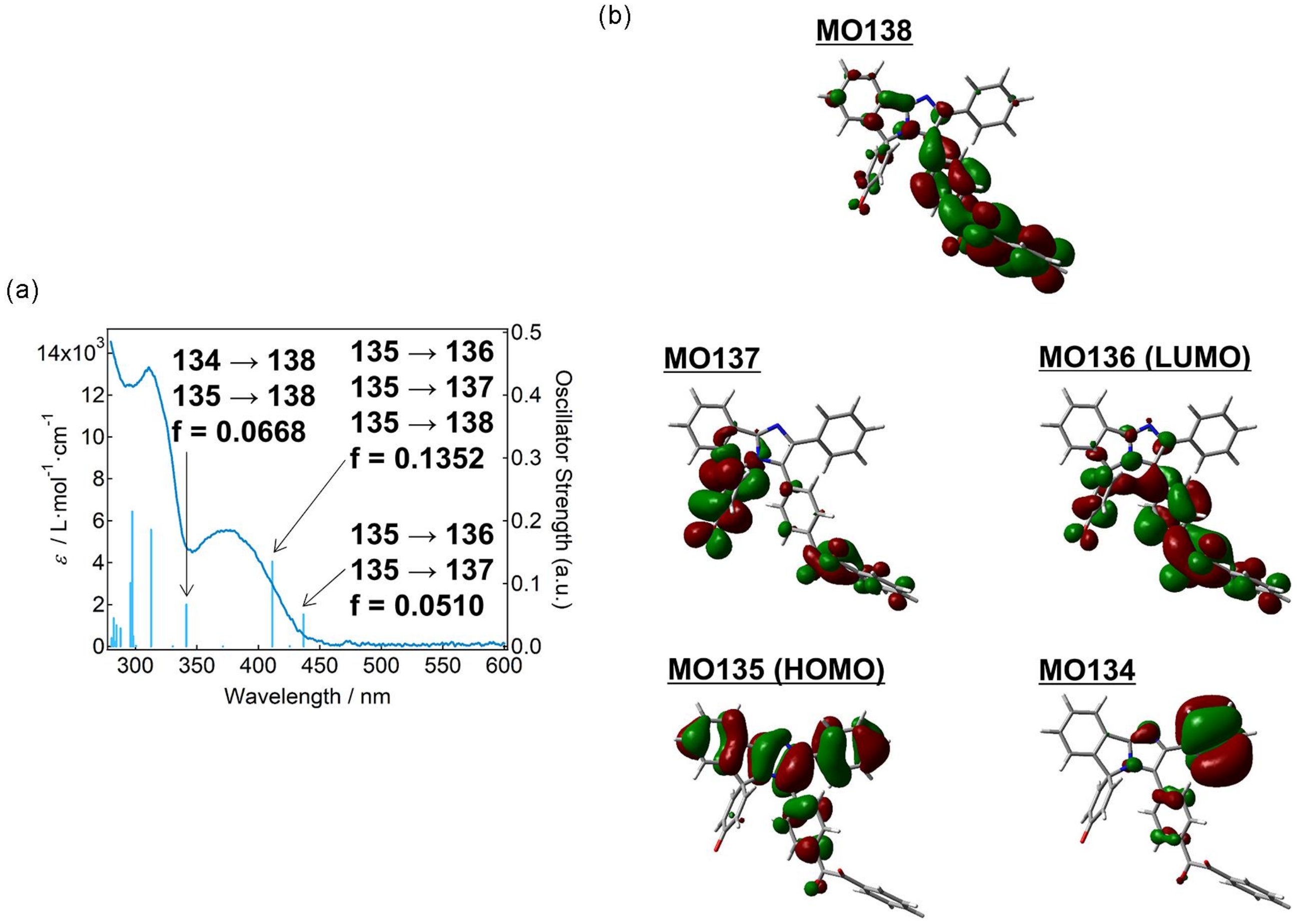

Figure S15. (a) UV–vis absorption spectrum of isomer B of Benzil-PIC in benzene. The calculated spectrum (MPW1PW91/6-31+G(d,p)//M05-2X/6-31+G(d,p) level of the theory) is shown by the vertical lines. (b) The relevant molecular orbitals of isomer B of Benzil-PIC calculated at the M05-2X/6-31+G(d,p) level of the theory.

9. Reference

S1. Frisch, M. J.; Trucks, G. W.; Schlegel, H. B.; Scuseria, G. E.; Robb, M. A.; Cheeseman, J. R.; Scalmani, G.; Barone, V.; Mennucci, B.; Petersson, G. A.; Nakatsuji, H.; Caricato, M.; Li, X.; Hratchian, H. P.; Izmaylov, A. F.; Bloino, J.; Zheng, G.; Sonnenberg, J. L.; Hada, M.; Ehara, M.; Toyota, K.; Fukuda, R.; Hasegawa, J.; Ishida, M.; Nakajima, T.; Honda, Y.; Kitao, O.; Nakai, H.; Vreven, T.; Montgomery, J. A., Jr.; Peralta, J. E.; Ogliaro, F.; Bearpark, M.; Heyd, J. J.; Brothers, E.; Kudin, K. N.; Staroverov, V. N.; Kobayashi, R.; Normand, J.; Raghavachari, K.; Rendell, A.; Burant, J. C.; Iyengar, S. S.; Tomasi, J.; Cossi, M.; Rega, N.; Millam, N. J.; Klene, M.; Knox, J. E.; Cross, J. B.; Bakken, V.; Adamo, C.; Jaramillo, J.; Gomperts, R.; Stratmann, R. E.; Yazyev, O.; Austin, A. J.; Cammi, R.; Pomelli, C.; Ochterski, J. W.; Martin, R. L.; Morokuma, K.; Zakrzewski, V. G.; Voth, G. A.; Salvador, P.; Dannenberg, J. J.; S. 50 Dapprich, S.; Daniels, A. D.; Farkas, Ö.; Foresman, J. B.; Ortiz, J. V.; Cioslowski, J.; Fox, D. J. Gaussian 09, Revision D.01; Gaussian, Inc.: Wallingford CT, 2009.
